# DNA ADP-Ribosylation Stalls Replication and Is Reversed by RecF-Mediated Homologous Recombination and Nucleotide Excision Repair

**DOI:** 10.1016/j.celrep.2020.01.014

**Published:** 2019-02-04

**Authors:** Emeline Lawarée, Gytis Jankevicius, Charles Cooper, Ivan Ahel, Stephan Uphoff, Christoph M. Tang

**Affiliations:** 1 Sir William Dunn School of Pathology, University of Oxford, Oxford OX1 3RE, UK; 2 Department of Biochemistry, University of Oxford, Oxford OX1 3QU, UK

**Keywords:** DNA ADP-ribosylation, toxin-antitoxin system, EPEC, DNA damage, SOS response, nucleotide excision repair

## Abstract

ADP-ribosylation of proteins is crucial for fundamental cellular processes. Despite increasing examples of DNA ADP-ribosylation, the impact of this modification on DNA metabolism and cell physiology is unknown. Here, we show that the DarTG toxin-antitoxin system from enteropathogenic *Escherichia coli* (EPEC) catalyzes reversible ADP-ribosylation of single-stranded DNA (ssDNA). The DarT toxin recognizes specific sequence motifs. EPEC DarG abrogates DarT toxicity by two distinct mechanisms: removal of DNA ADP-ribose (ADPr) groups and DarT sequestration. Furthermore, we investigate how cells recognize and deal with DNA ADP-ribosylation. We demonstrate that DNA ADPr stalls replication and is perceived as DNA damage. Removal of ADPr from DNA requires the sequential activity of two DNA repair pathways, with RecF-mediated homologous recombination likely to transfer ADP-ribosylation from single- to double-stranded DNA (dsDNA) and subsequent nucleotide excision repair eliminating the lesion. Our work demonstrates that these DNA repair pathways prevent the genotoxic effects of DNA ADP-ribosylation.

## Highlights

• Enteropathogenic *E. coli* DarTG toxin:antitoxin reversibly ADP-ribosylates ssDNA
• DNA ADP-ribosylation stalls replication and can be repaired by two pathways
• RecF-mediated homologous recombination is likely to transfer the lesion to dsDNA
• Subsequent nucleotide excision repair eliminates the lesion from DNA

## Graphical Abstract

**Figure:**
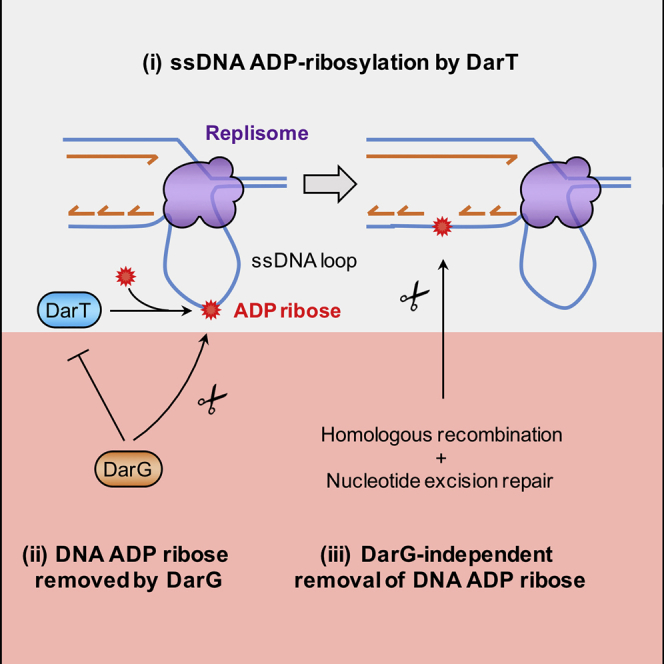

## Teaser

Little is known about how cells sense and remove DNA ADP-ribosylation. Lawarée et al. demonstrate that the enteropathogenic *E. coli* DarTG toxin:antitoxin reversibly ADP-ribosylates ssDNA, stalling DNA replication. Cells recognize DNA ADP-ribosylation as damage and repair this lesion by the sequential activity of RecF-mediated homologous recombination and nucleotide excision repair.

## Introduction

ADP-ribosylation is a reversible post-translational modification found in all domains of life (Aravind et al., 2015). In eukaryotes, protein ADP-ribosylation has been studied extensively and influences fundamental processes such as transcription, cell division, metabolism, and DNA repair (Barkauskaite et al., 2015, Gibson and Kraus, 2012, Perina et al., 2014). This post-translational modification is catalyzed by mono-ADP-ribosyl transferases (ARTs) or poly-ADP-ribose polymerases (PARPs) that transfer single or multiple ADP-ribose (ADPr) group or groups, respectively, from nicotinamide adenine dinucleotide (NAD^+^) onto target proteins. ADPr groups are removed from proteins by glycohydrolases, which usually contain a macrodomain (Rack et al., 2016). In contrast, few prokaryotic ARTs have been characterized, most of which are secreted enzymes that contribute to virulence by targeting host proteins. For instance, cholera toxin is secreted by *Vibrio cholerae* and inactivates G proteins in intestinal epithelial cells (Holmgren et al., 1975), while diphtheria toxin prevents protein translation by ADP-ribosylation of EF-2, leading to host cell death (Strauss and Hendee, 1959).

It is becoming increasingly appreciated that ADP-ribosylation is not restricted to proteins but can also affect other macromolecules such as tRNAs (Spinelli et al., 1999), small-molecule antimicrobials (Dabbs et al., 1995), and more recently DNA (Jankevicius et al., 2016, Talhaoui et al., 2016, Nakano et al., 2006). The first description of DNA ADP-ribosylation involved pierisins produced by cabbage butterfly larvae, which ADP-ribosylate 2′-deoxyguanosines in double-stranded DNA (dsDNA), causing apoptotic cell death (Takamura-Enya et al., 2001). Other mammalian ARTs can modify ends of DNA and RNA *in vitro* (Munnur and Ahel, 2017, Munnur et al., 2019, Talhaoui et al., 2016). However, it is not clear whether this modification occurs *in vivo* or what the consequences are of DNA ADP-ribosylation.

In prokaryotes, the only example of DNA ADP-ribosylation is a toxin-antitoxin (TA) system from *Thermus aquaticus*, which harbors a toxin DarT that ADP-ribosylates single-stranded DNA (ssDNA) (Jankevicius et al., 2016), in contrast to mammalian PARPs, which target dsDNA (Jankevicius et al., 2016, Takamura-Enya et al., 2001). Overexpression of *T. aquaticus darT* in *Escherichia coli* inhibits growth by stalling DNA replication, while the antitoxin DarG removes the ADPr group from ssDNA (Jankevicius et al., 2016). DarTG is widespread in bacteria, including pathogenic bacteria such as enteropathogenic *Escherichia coli* (EPEC), *Mycobacterium tuberculosis*, and *Klebsiella pneumoniae*. Therefore, although DNA ADP-ribosylation occurs in eukaryotes and prokaryotes, the cellular response to and repair of this modification remain poorly understood.

Here we characterized *E. coli* DarTG and examined its impact on DNA metabolism. We found that DarTG from EPEC is a functional TA system. Although EPEC DarT shares many features of *T. aquaticus* DarT, we show that it targets a different DNA sequence motif. Furthermore, we demonstrate that DarG has two distinct mechanisms to counteract DarT toxicity: enzymatic removal of DNA ADP-ribosylation via its N-terminal macrodomain and physical sequestration of DarT via its C-terminal domain. We also show that DNA ADP-ribosylation stalls DNA replication and leads to an increase in RecA levels, indicating that cells perceive this modification as DNA damage. Activation of TA systems usually occurs via loss of antitoxin activity. Without DarG, cells must rely on other mechanisms to survive DNA damage caused by DarT. Indeed, we identified a pathway that allows recognition and reversal of DNA ADP-ribosylation, independent of DarG. RecF-mediated homologous recombination (HR) converts ADP-ribosylated ssDNA into a dsDNA lesion, which is then removed by the nucleotide excision repair (NER) pathway. In summary, our data demonstrate the impact of DNA ADP-ribosylation on DNA metabolism, illustrate how EPEC perceives this DNA modification, and reveal the mechanisms that remove ADPr groups from DNA.

## Results

### DarT Is a Toxin that ADP-Ribosylates ssDNA and Stalls DNA Replication

**Figure 1:**
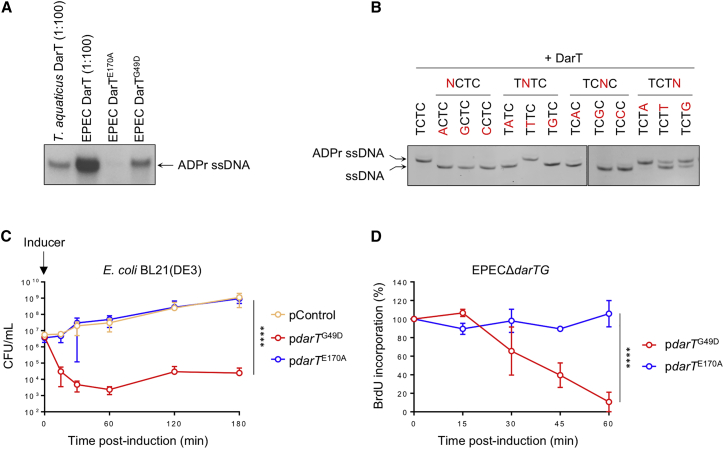
EPEC DarT ADP-Ribosylates ssDNA, Reducing DNA Replication and Bacterial Viability (A) ADP-ribosylation of 27-mer ssDNA oligonucleotide (ADPr-EL1, sequence in Table S1) incubated with purified *T. aquaticus* DarT, EPEC DarT, DarT^E170A^, or DarT^G49D^ with ^32^P-NAD^+^; n = 3, data from one experiment shown; 1:100 = 100 times diluted. (B) ADP-ribosylation of 27-mer ssDNA oligonucleotide incubated with purified EPEC DarT and NAD^+^. ADPr ssDNA, ADP-ribosylated ssDNA. The oligonucleotide sequences used in the assays are listed in Table S1; n = 3, representative data from one experiment shown. (C) Viability of *E. coli* BL21(DE3) following expression of *darT*^G49D^ or *darT*^E170A^. pControl, empty vector; n = 3 ± SD, ^∗∗∗∗^p < 0.0001 by two-way ANOVA. (D) Percentage of BrdU incorporation in EPECΔ*darTG* following expression of *darT*^G49D^ or *darT*^E170A^; n = 3 ± SD, ^∗∗∗∗^p < 0.0001 by two-way ANOVA.

Previous work demonstrated that *T. aquaticus* DarT ADP-ribosylates ssDNA (Jankevicius et al., 2016). Because of the level of sequence similarity between *T. aquaticus* and EPEC DarT (29.5% identity, Figure S1A) and lack of knowledge of its mechanism of action, we assessed whether EPEC DarT can ADP-ribosylate ssDNA. EPEC DarT was produced by *in vitro* transcription/translation (Figure S1B) and then incubated with a ssDNA oligonucleotide containing the sequence TCTC (corresponding to the TNTC motif recognized by *T. aquaticus* DarT) and ^32^P-NAD^+^ as the co-factor. In the presence of DarT, the oligonucleotide was radiolabeled, demonstrating that EPEC DarT is a ssDNA ART similar to the *T. aquaticus* enzyme (Figures 1A and S1B, showing the levels of proteins used in assays). Alanine substitution of a conserved glutamate crucial for the ART activity renders *T. aquaticus* DarT inactive (Jankevicius et al., 2016). Similarly, substitution of the corresponding glutamic acid in EPEC DarT (DarT^E170A^, Figure S1A) leads to barely detectable DNA ADP-ribosylation (Figure 1A). In contrast, we found no evidence that wild-type EPEC DarT ADP-ribosylates dsDNA (Figure S2A). To determine whether EPEC DarT also recognizes a specific sequence, we next performed non-radioactive ADP-ribosylation assays with purified EPEC DarT and ssDNA oligonucleotides containing various substitutions in the TCTC sequence. In contrast to *T. aquaticus*, we found that EPEC DarT preferentially ADP-ribosylates the sequences TTT or TCT (Figures 1B and S2B).

To determine the function of EPEC DarT *in vivo*, we attempted to express wild-type *darT* from a low copy plasmid under the control of a repressible promoter. This approach proved unsuccessful, probably because of the lethal effect of DarT. Instead, we isolated a mutated version of DarT containing a single point mutation (*darT*^G49D^) and found that this modified toxin retains ssDNA ART activity, albeit to a lesser extent than the wild-type protein (Figure 1A); the altered glycine residue is not conserved in *T. aquaticus* DarT (Figure S1A). Expression of *darT*^G49D^ also decreased the viability of EPEC lacking *darTG* (EPECΔ*darTG*) and *E. coli* BL21(DE3) by approximately one and four orders of magnitude, respectively, while the inactive toxin DarT^E170A^ had no effect on bacterial survival (Figures 1C and S3A, p < 0.0001 for both strains). The reduced toxicity of DarT^G49D^ in EPEC compared with *E. coli* BL21(DE3) is likely to be a consequence of its lower expression following arabinose induction in EPEC (Figure S3B).

Because DarT modifies ssDNA, we hypothesized that it may cause toxicity by hindering DNA replication. DNA replication generates ssDNA loops of the lagging strand template, which could be a substrate for DarT (Langston and O’Donnell, 2006). To assess the progression of replication, we measured incorporation of the thymidine analog, bromodeoxyuridine (BrdU), into nascent DNA after inducing the expression of *darT*^G49D^ or *darT*^E170A^ in EPECΔ*darTG* (Urbach et al., 1999). Although expression of inactive *darT*^E170A^ did not affect DNA replication, *darT*^G49D^ expression led to a marked reduction of BrdU incorporation within 45 min in EPEC (p < 0.0001, Figure 1D), indicating that active DarT impedes DNA replication. Furthermore, BrdU incorporation decreased within 5 min of expressing *darT*^G49D^ in *E. coli* BL21(DE3) (Figure S3C), consistent with this strain’s sensitivity to DarT (Figures 1C and S3A).

### DarG Counteracts DarT Toxicity by Two Distinct Mechanisms

**Figure 2:**
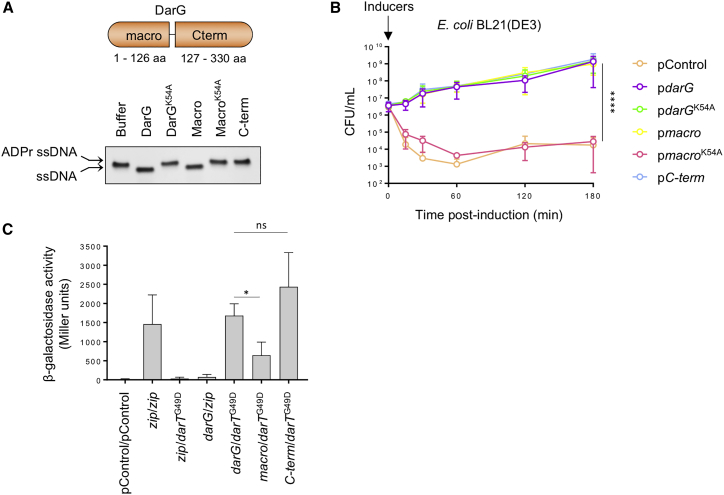
DarG Counteracts DarT^G49D^ Toxicity (A) De-ADP-ribosylation of a 27-mer ADP-ribosylated ssDNA oligonucleotide incubated with purified EPEC DarG, DarG^K54A^, the macrodomain (Macro) of DarG, Macrodomain^K54A^ (Macro^K54A^), or the C-terminal domain (C-term) of DarG. ADPr ssDNA, ADP-ribosylated ssDNA; n = 3, data from one experiment shown. (B) Viability of *E. coli* BL21(DE3) following expression of *darT*^G49D^ with different versions of *darG*. pControl, empty plasmid; n = 3 ± SD, ^∗∗∗∗^p < 0.0001 by two-way ANOVA. (C) Bacterial two-hybrid assay of *E. coli* BTH101 containing pUT18 expressing T18 fused to *zip*, *darG*, *macro*, or *C-term* and pKT25 expressing T25 fused to *zip* or *darT*^G49D^. pControl, empty pUT18 or pKT25; Zip, Zipper leucine, used as a positive control; ns, not significant; n = 3 ± SD, ^∗^p < 0.05 by Student’s t test.

Next, we assessed the glycohydrolase activity of DarG using de-ADP-ribosylation assays. Incubation of ADP-ribosylated ssDNA with purified DarG (Figure S1C) resulted in removal of the DNA modification (Figure 2A), demonstrating that DarG can eliminate ADPr from ssDNA, similar to *T. aquaticus* DarG (Jankevicius et al., 2016). Furthermore, we investigated whether EPEC DarG can prevent DarT toxicity *in vivo* by introducing *darG* under the control of an isopropyl β-D-1-thiogalactopyranoside (IPTG)-inducible promoter, with *darT*^G49D^ under the control of an arabinose-inducible promoter in *E. coli* BL21(DE3). The reduction in bacterial viability because of *darT*^G49D^ expression was prevented by co-expression of *darG* (p < 0.001), confirming that EPEC DarTG is a functional TA system (Figure 2B).

A lysine residue in *T. aquaticus* DarG (K80) is present in the ADPr binding site of its macrodomain and is essential for glycohydrolase activity (Jankevicius et al., 2016). Alanine substitution of the corresponding residue in EPEC DarG (generating DarG^K54A^) leads to undetectable DNA ADP-ribosyl glycohydrolase activity (Figures 2A and S1A). However, surprisingly, we found that DarG^K54A^ still prevents DarT^G49D^ toxicity *in vivo* (Figure 2B), suggesting that EPEC DarG can counteract DarT toxicity via a mechanism distinct from its glycohydrolase activity.

Therefore, to further characterize DarG, we examined the functions of its N-terminal macrodomain (aa 1–126) and its C-terminal domain (aa 127–330) independently (Figure S1A). Initially, we found that the DarG macrodomain is sufficient to both de-ADP-ribosylate ssDNA *in vitro* (Figure 2A) and abrogate DarT^G49D^ toxicity *in vivo* (Figure 2B), while K54A substitution of the DarG macrodomain abolishes DNA ADP-ribosyl glycohydrolase activity (Figure 2A) and is incapable of restoring bacterial viability in the presence of DarT^G49D^ (Figure 2B). Intriguingly, although the C-terminal domain of DarG lacks detectable DNA ADP-ribosyl glycohydrolase activity (Figure 2A), it is sufficient to counteract DarT^G49D^ toxicity *in vivo* (Figure 2B). Because antitoxins of type II TA systems act by sequestering toxin proteins, we next performed a bacterial two-hybrid assay to assess whether the C-terminal domain of DarG directly interacts with DarT (Karimova et al., 2000). Therefore, we fused the T18 and T25 domains of *Bordetella pertussis* adenylate cyclase to full-length or truncated *darG*, and *darT*^G49D^, respectively, and quantified interactions by measuring β-galactosidase activity (Karimova et al., 2000). DarG showed a significant level of interaction with DarT (Figure 2C). The C-terminal domain of DarG alone was sufficient for interaction (p = 0.2403), whereas binding of the N-terminal macrodomain of DarG was significantly weaker (p = 0.0318, Figure 2C), indicating that EPEC DarG directly interacts with DarT^G49D^, predominantly through its C-terminal domain.

Altogether, these results demonstrate that EPEC DarT ADP-ribosylates ssDNA at the sequence TCT or TTT, impairing DNA replication and bacterial survival. DarG counteracts DarT^G49D^ toxicity via both its macrodomain (by reversing DNA ADP-ribosylation) and its C-terminal domain (by sequestering the toxin).

### The SOS Response Counters DarT-Mediated Toxicity

Next, we determined whether DNA ADP-ribosylation is perceived as DNA damage, because inhibiting DNA replication should lead to activation of the SOS DNA damage response, which is triggered when RecA polymerizes on ssDNA at DNA breaks or stalled replication forks (Defais et al., 1971, Witkin, 1976). Activated RecA catalyzes the auto-proteolysis of the transcriptional repressor LexA (Little, 1991), allowing expression of more than 40 genes involved in the SOS response, including those encoding RecA and other DNA repair and damage tolerance genes (Courcelle et al., 2001, Fernández De Henestrosa et al., 2000, Kenyon and Walker, 1980, Lewis et al., 1994, Little and Mount, 1982).

**Figure 3:**
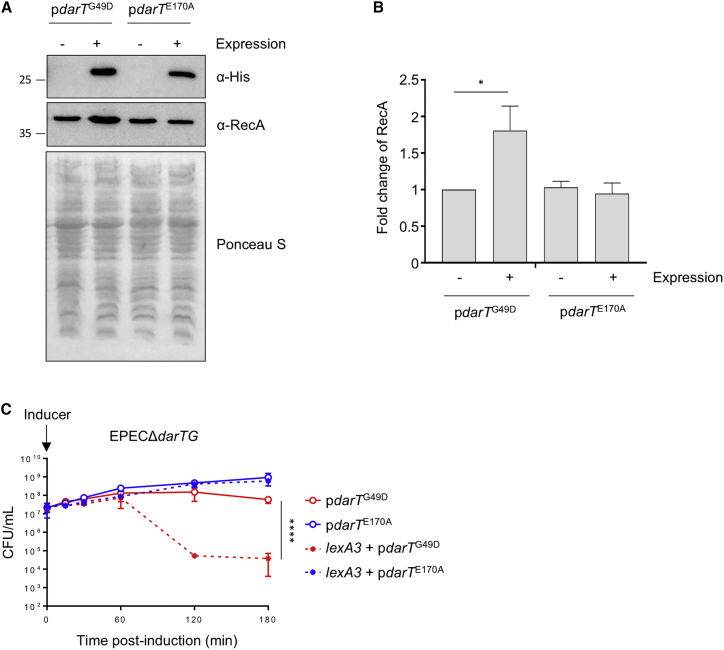
The SOS Response Reduces DarT^G49D^ Toxicity (A) Levels of His-tagged DarT^G49D^ or DarT^E170A^ and RecA detected by western blot analysis of *E. coli* BL21(DE3) cell lysates. Expression of His-DarT^G49D^/His-DarT^E170A^ was induced for 3 h with 0.8% arabinose. Ponceau S is shown as a loading control. (B) Quantification of RecA levels following expression of *darT*^G49D^ or *darT*^E170A^; n = 3 ± SD, ^∗^p < 0.05 by Student’s t test. (C) Viability of EPEC*ΔdarTG* or EPEC*ΔdarTG_lexA3* following expression of *darT*^G49D^ or *darT*^E170A^; n = 3 ± SD, ^∗∗∗∗^p < 0.0001 by two-way ANOVA.

Therefore, we next investigated whether expression of *darT* increases cellular RecA levels, a hallmark of the SOS response. Although there was no increase in RecA abundance following expression of inactive *darT*^E170A^, overexpression of *darT*^G49D^ significantly increased RecA levels (p = 0.0139, Figures 3A and 3B). To test whether the SOS response contributes to survival following expression of *darT*^G49D^, we generated an EPEC strain expressing an uncleavable version of LexA (LexA3), which is unable to mount an SOS response (Figure S4) (Little et al., 1980); this strain was significantly more sensitive to *darT*^G49D^ expression compared with the strain with wild-type *lexA* (p < 0.0001, Figure 3C). These results demonstrate that ADP-ribosylation of DNA is recognized as a form of DNA damage, with the SOS response reducing DarT^G49D^ toxicity and allowing bacterial growth following DNA ADP-ribosylation.

### RecF-Mediated Homologous Recombination Prevents Toxicity due to DNA ADP-Ribosylation

**Figure 4:**
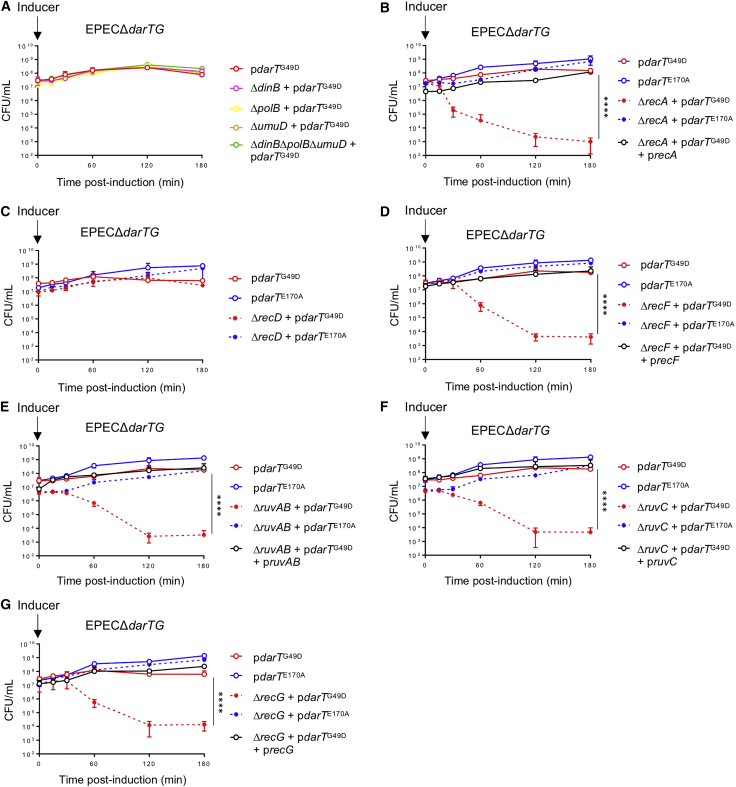
RecFOR-HR Pathway and Processing of Holliday Junction Intermediates Prevent DarT^G49D^-Mediated Toxicity (A–G) Viability of mutants following expression of *darT*^G49D^ or *darT*^E170A^ to examine the role of (A) the translesion polymerases encoded by *dinB*, *polB*, and *umuD*; (B) RecA; (C) RecD; (D) the RecFOR pathway; or enzymes processing Holliday junction intermediates: (E) *ruvAB*, (F) *ruvC*, and (G) *recG*. The *recA*, *recF*, *ruvAB*, *ruvC*, and *recG* mutants were complemented by providing the gene on a low copy number plasmid; n = 3 ± SD, ^∗∗∗∗^p < 0.0001 by two-way ANOVA.

To elucidate the mechanisms by which bacteria circumvent DarT toxicity, we examined DNA repair and DNA damage tolerance genes in the LexA regulon. In *E. coli*, LexA regulates expression of the translesion DNA polymerase (Pol) II (encoded by *polB*), Pol IV (*dinB*), and Pol V (*umuDC*) that allow DNA replication through sites of damage that cannot be bypassed by replicative DNA Pol III (Goodman and Woodgate, 2013). However, deletion of *polB*, *dinB*, and *umuDC*, either singly or in combination, did not enhance DarT^G49D^ toxicity in EPECΔ*darTG* (p > 0.4118), suggesting that the translesion polymerases do not contribute to survival in the presence of DNA ADP-ribosylation (Figure 4A).

In contrast, we found that RecA plays a major role in preventing DarT^G49D^ toxicity, because the survival of EPEC lacking *recA* is significantly lower following the expression of the toxin compared with the isogenic *recA*^+^ strain (p < 0.0001, Figure 4B). Indeed, the *recA* deletion strain was even more sensitive than the *lexA3* strain, indicating that RecA has a central function in survival following DNA ADP-ribosylation, in addition to its role in the SOS response. The activity of RecA is regulated by binding RecFOR or the RecBCD complex, which initiate the repair of ssDNA gaps (SSGs) or dsDNA breaks, respectively (Spies and Kowalczykowski, 2005). Therefore, we constructed strains to disrupt each of these pathways. Because multiple attempts to generate EPEC *recB*, *recC*, or *recBC* mutants were unsuccessful, we constructed a *recD* mutant, although RecD is not essential for RecBC-mediated recombination (Lovett et al., 1988). Although the *recD* mutant does not exhibit increased DarT^G49D^ toxicity (p = 0.9280, Figure 4C), EPECΔ*darTG* lacking *recF* has significantly reduced survival following *darT*^G49D^ expression (p < 0.0001, Figure 4D), indicating that the RecFOR pathway is involved in the tolerance of cells to DNA ADP-ribosylation.

During repair of SSGs, RecFOR binds to the 5′ end of ssDNA-dsDNA junctions and recruits RecA (Morimatsu and Kowalczykowski, 2003). RecA nucleoprotein filaments associated with ssDNA search for and invade homologous dsDNA, resulting in DNA strand exchange and formation of Holliday junctions (HJs). Next, the RuvAB complex or RecG helicase catalyze HJ branch migration, and finally, RuvC resolves the HJ to produce two recombinant dsDNAs (West, 1996). Consistent with results for the *recF* mutant, loss of *ruvAB*, *ruvC*, or *recG* also significantly reduces the survival of EPECΔ*darTG* following expression of *darT*^G49D^ (p < 0.0001 for each mutant compared with bacteria with each gene), while complementation of the mutants restores survival to wild-type levels (Figures 4E–4G). Altogether, these data indicate that ADP-ribosylation of ssDNA generates SSGs that can be repaired by the RecFOR-mediated HR pathway and resolved by RuvC.

### NER Contributes to Survival following DarT^G49D^ Expression

**Figure 5:**
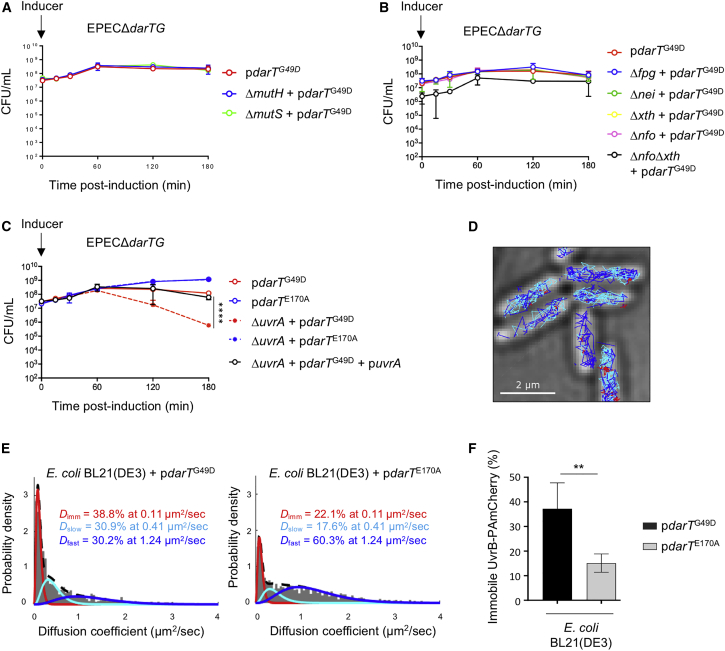
Recruitment of the NER Pathway in Response to DNA ADP-Ribosylation (A–C) Viability of EPEC*ΔdarTG* lacking single members of (A) the MMR pathway (EPEC*ΔdarTGΔmutH* and EPEC*ΔdarTGΔmutS*), (B) the BER pathway (EPEC*ΔdarTGΔfpg* and EPEC*ΔdarTGΔnei*, EPEC*ΔdarTGΔxth*, and EPEC*ΔdarTGΔnfo*), and (C) the NER pathway (EPEC*ΔdarTGΔuvrA*) following expression of *darT*^G49D^; n = 3 ± SD, ^∗∗∗∗^p < 0.00001 by two-way ANOVA with or without *uvrA* + p*darT*^G49D^. (D) Single-molecule tracking of UvrB-PAmCherry over 10,000 frames (with a 15 ms interval between frames) following expression of *darT*^G49D^. Immobile UvrB-PAmCherry molecules with *D*^∗^ < 0.2 μm^2^/s are in red, with 0.2 μm^2^/s < *D*^∗^ (the diffusion coefficient) < 1.5 μm^2^/s in turquoise and *D*^∗^ > 1.5 μm^2^/s in blue. (E) Determination of *D*^∗^ values of UvrB-PAmCherry in *E. coli*, fitted with a three-species model following 15 min expression of *darT*^G49D^ or *darT*^E170A^. (F) Percentage of immobile UvrB-PAmCherry in *E. coli* after 15 min expression of *darT*^G49D^ or *darT*^E170A^; n = 4 biological replicates ± SD, total number of cells = 14,338, total number of tracks = 142,748, ^∗∗^p < 0.01 by unpaired t test (two tailed).

RecF-mediated HR followed by HJ resolution would not remove ADP-ribosylation from DNA but instead would transfer the modification from ssDNA to dsDNA (Spies and Kowalczykowski, 2005). In *E. coli*, dsDNA lesions are recognized by three main cellular pathways: mismatch repair (MMR), base excision repair (BER), and NER. To investigate whether MMR counteracts DNA ADP-ribosylation, we measured the survival of EPECΔ*darTG* lacking either *mutS* or *mutH*, essential for the recognition or excision of DNA lesions by MMR, respectively, following expression of *darT*^G49D^. We found that there was no increase of DarT^G49D^ toxicity in strains lacking *mutS* or *mutH* (p > 0.9363), suggesting MMR does not counter the toxicity of DNA ADP-ribosylation (Figure 5A).

Because of the redundancy of BER, it is difficult to inactivate this pathway (Krokan and Bjørås, 2013). Therefore, we generated mutants lacking major DNA glycosylases (Fpg and Nei) or AP (apurinic/apyrimidinic) endonucleases (Xth and Nfo) involved in BER. All mutants exhibit sensitivity to DarT^G49D^ similar to that of EPECΔ*darTG* (p > 0.9859), providing no evidence that BER repairs DNA ADP-ribosylation (Figure 5B). Given the importance of AP endonucleases in BER, we also generated a strain lacking both *xth* and *nfo*, which encode the two members of this family of enzymes in *E. coli* (Krokan and Bjørås, 2013). Again, there was no increase in the sensitivity of this mutant to DarT^G49D^ expression.

NER is regulated by the SOS response and removes a range of lesions that cause structural distortion of the DNA helix (Kisker et al., 2013). UvrA localizes to sites of DNA damage and recruits UvrB to a lesion (Stracy et al., 2016). UvrB then directs the excision and removal of damaged ssDNA by UvrC and UvrD, respectively; the remaining SSG is filled by DNA Pol I and repaired by ligase (Kisker et al., 2013). To determine whether NER is involved in preventing DarT^G49D^ toxicity, we constructed a mutant lacking *uvrA*, which is essential for NER. EPECΔ*darTG*Δ*uvrA* has a significant decrease in survival after *darT*^G49D^ expression, compared with the isogenic *uvrA*^+^ strain (p < 0.0001, Figure 5C), while complementation of the mutant strain reverses the defect (Figure 5C).

To provide independent evidence that NER is activated following *darT*^G49D^ expression, we assessed the motility of UvrB in single-molecule tracking experiments of *E. coli* BL21(DE3) grown in M9 medium; expression of toxin *darT*^G49D^ reduces bacterial survival in this medium (Figure S3D). Photoactivated localization microscopy (PALM) can be used to follow the fate of individual fluorescently labeled DNA repair proteins one at a time as they search for and repair DNA lesions in live cells (Uphoff et al., 2013). After UvrA recognizes DNA damage, UvrB is recruited to DNA and becomes immobile. Hence, the relative abundance of mobile and immobile UvrB molecules provides a direct readout for the activity of the NER pathway (Stracy et al., 2016). We used an endogenous allele of *uvrB* fused at its C terminus to PAmCherry, a photoactivable fluorescent protein (Stracy et al., 2016). We confirmed that the fusion protein is functional, because there was no difference in viability between the wild type and the strain containing chromosomal UvrB-PAmCherry following UV exposure or *darT*^G49D^ expression, whereas a Δ*uvrB* deletion strain was significantly more sensitive (Figures S5A and S5B). In the absence of exogenous stress, the motility of UvrB-PAmCherry molecules can be summarized by fitting the observed distribution of diffusion coefficients with a model containing three molecular species: immobile (motility 0.11 μm^2^/s), slowly diffusing (0.41 μm^2^/s), and fast-diffusing molecules (1.24 μm^2^/s, Figures 5D and 5E), matching previous results (Stracy et al., 2016). Strikingly, following expression of *darT*^G49D^, 35% of the total UvrB-PAmCherry pool became immobile, compared with only 16% after non-functional *darT*^E170A^ was expressed in cells (p = 0.0043, Figure 5F), confirming that UvrB is recruited to DNA in the presence of DNA ADP-ribosylation. We confirmed that this recruitment was specific to the activity of UvrB in the NER pathway, because there was no change in UvrB-PAmCherry motility in a *uvrA*^−^ strain (which is unable to initiate NER) following expression of *darT*^G49D^ (Figures S5D–S5E).

**Figure 6:**
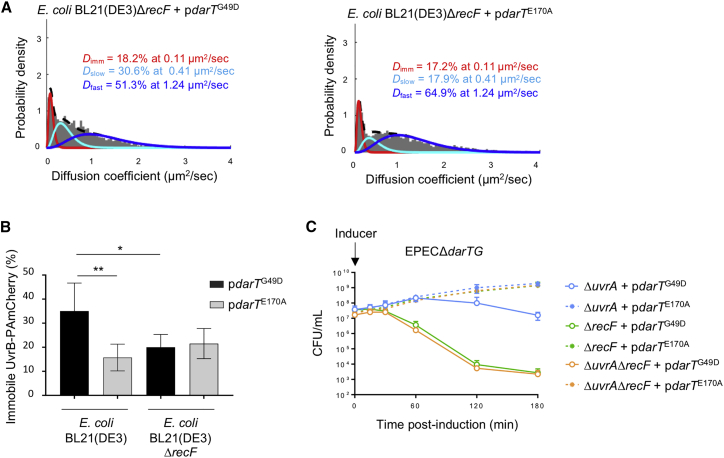
Repair of DNA ADP-Ribosylation by Sequential Action of RecF-Mediated HR followed by NER (A) Determination of *D*^∗^ values of UvrB-PAmCherry in *E. coli* BL21(DE3)Δ*recF*, fitted with a three-species model (three-constraint fit) following 15 min expression of *darT*^G49D^ or *darT*^E170A^. (B) Percentage of immobile UvrB-PAmCherry in *E. coli* BL21(DE3) and *E. coli* BL21(DE3)Δ*recF* after 15 min expression of *darT*^G49D^ or *darT*^E170A^; 10,000 frames per movie, with a 15 ms interval between frames; n = 5 biological replicates ± SD, total number of cells = 23,031, total number of tracks = 219,672; ^∗^p < 0.05, ^∗∗^p < 0.01 by unpaired t test (two tailed). (C) Viability of EPEC*ΔdarTGΔuvrA*, EPEC*ΔdarTGΔrecF*, and EPEC*ΔdarTGΔuvrAΔrecF* following expression of *darT*^G49D^; n = 3 biological replicates ± SD, NS = p > 0.9999 by two-way ANOVA between EPEC*ΔdarTGΔrecF + pdarT*^G49D^ and EPEC*ΔdarTGΔuvrAΔrecF* + p*darT*^G49D^.

**Figure 7:**
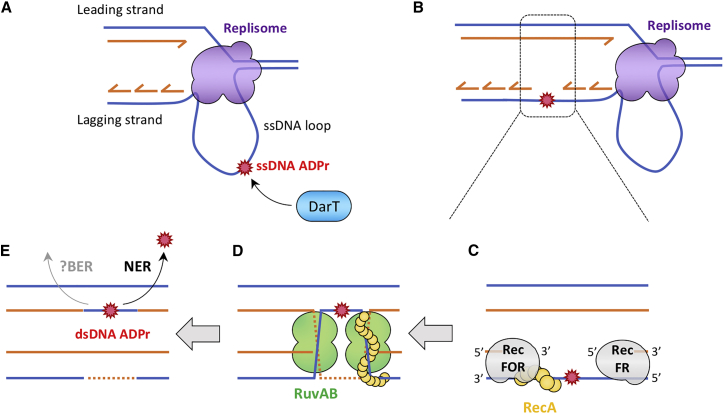
Model: EPEC DarT ADP-Ribosylates ssDNA and Stalls DNA Replication (A) DNA replication generates ssDNA loops, potential substrates for DarT. (B) Toxicity due to the presence of ADPr on ssDNA loops is unaffected by translesion polymerases and might lead to the generation of SSGs after DNA replication. (C) RecFOR binds both extremities of the SSG and facilitates RecA nucleoprotein formation on ssDNA. RecA polymerization induces the SOS response and catalyzes the strand migration of the ssDNA into the homologous dsDNA, resulting in the formation of two Holliday junctions that are stabilized by RuvAB. (D) Junctions are then resolved by RuvC or RecG, generating two dsDNA molecules, with one of them containing dsDNA ADPr. (E) NER pathway is recruited in a RecF-dependent manner to deal with the dsDNA ADPr, although BER might also contribute.

Our finding that DNA ADP-ribosylation lesions are repaired by NER appears to be at odds with the observation that DarT targets ssDNA substrates. How do ssDNA lesions become embedded in dsDNA, where they can be recognized by NER? It is unlikely that ssDNA lesions are transferred to dsDNA simply by DNA synthesis, considering that DarT expression inhibits DNA replication (Figure 1D) and translesion polymerases do not contribute to DarT tolerance (Figure 4A). Instead, we hypothesized that RecFOR initiates HR of SSGs containing ADP-ribosylation lesions, given the role of HR in reducing DarT toxicity (Figures 4B and 4D). Subsequent HJ branch migration could transfer the lesion from ssDNA to dsDNA without the need for DNA synthesis to bypass the bulky lesion. To test this, we measured the motility of UvrB-PAmCherry in a strain lacking *recF*. In contrast to bacteria with *recF* (Figure 5F), there was no significant increase of immobile UvrB-PAmCherry in a *recF*^−^ strain following expression of *darT*^G49D^ (Figure 6B). As a control, exposure to UV light in the absence of *recF* still leads to a significant increase of immobile UvrB-PAmCherry (Figure S6), confirming that NER activity is independent of RecF following UV light exposure, but not after expression of DarT. Moreover, deletion of *uvrA* results in no additional reduction in bacterial survival following expression of *darT*^G49D^ in strains lacking *recF*, *ruvAB*, *ruvC*, or *recG* (Figures 6C and S7), providing genetic evidence that NER and RecFOR/RuvC operate in the same pathway to circumvent toxicity of DNA ADP-ribosylation (Figure 7).

## Discussion

Here we demonstrate that EPEC DarTG is a functional TA system that shares a mode of action similar to that of *T. aquaticus* DarTG. Like *T. aquaticus* DarT, the EPEC toxin is an ART that modifies ssDNA. However, the sequence specificity of the toxins differs slightly, with the EPEC recognition sequence involving three nucleotides (TTT/TCT), compared with the TNTC motif recognized by *T. aquaticus* DarT. Even if the toxins target different sequences, the two thymidines in the motif are conserved, suggesting that EPEC DarT modifies the second thymidine, as *T. aquaticus* DarT (Jankevicius et al., 2016), and that these two nucleotides are critical for the ability of DarT to recognize and interact with DNA. We also noticed that EPEC DarT is considerably more active *in vitro* than the *T. aquaticus* toxin (Figures 1A and S1B for the levels of proteins in assays). Sequence alignments do not offer any obvious reason for these differences, although the atomic structure of DarT in a complex with its substrate might provide insights. It is also important to consider that *T. aquaticus* is an extremophile and its enzymes are optimized for temperatures higher than 37°C, the temperature we used for *in vitro* ADP-ribosylation assays.

Similar to *T. aquaticus* DarG, EPEC DarG counteracts DarT-mediated toxicity by removing ADPr groups from DNA via its macrodomain, acting as a type IV TA system (Jankevicius et al., 2016), and could be considered a DNA repair enzyme, because it removes adducts from DNA. Another example of an enzyme that directly removes nucleotidylated DNA adducts (AMP, in this case) is Aprataxin (Ahel et al., 2006). In addition, we found that the C-terminal domain of EPEC DarG can prevent DarT toxicity by sequestering the toxin. The DarG-DarT interaction might prevent DarT from accessing its target, as in type II TA systems (Yamaguchi and Inouye, 2011).

Although protein ADP-ribosylation was described more than 50 years ago, studies of this modification on DNA are still in their infancy, with reports limited to *in vitro* descriptions of the addition of ADPr to dsDNA (Takamura-Enya et al., 2001) or on dsDNA breaks (Munnur and Ahel, 2017, Talhaoui et al., 2016). Hence, the consequences of DNA ADP-ribosylation *in vivo* have been largely unexplored. Therefore, a key focus of our work was to define the cellular processes affected by DNA ADP-ribosylation and to understand how this modification is recognized and dealt with.

Expression of DarT led to the inhibition of DNA replication (Figure 1D for EPEC), which occurred within 5 min in *E. coli* BL21(D3) (Figure S3C) and could be caused by DNA ADP-ribosylation stalling DNA synthesis. However, we cannot exclude the possibility that DarT affects other processes, such as transcription, that also generate ssDNA loops (Gowrishankar et al., 2013). The contribution of the SOS response in reversing DNA ADP-ribosylation was evident from the elevated RecA expression (Figures 3A and 3B) and the significant increase of DarT^G49D^ toxicity in an EPEC strain that is unable to elicit the SOS response (i.e., expressing *lexA3*, Figure 3C).

The ability to complete DNA replication is essential for genomic integrity. During DNA replication, cells must overcome different replication fork barriers that can be mutagenic or lethal if not repaired. Intrinsic replication fork barriers include DNA binding proteins, transcription, or unusual DNA structures (e.g., quadruplex DNA) (Mirkin and Mirkin, 2007), but barriers can also be derived from exogenous sources (Courcelle et al., 2003, Cox, 2001). Two main pathways are employed to restart DNA replication after replication forks have been blocked by DNA lesions (Costes and Lambert, 2012, Goodman and Woodgate, 2013). Translesion polymerases, also known as error-prone polymerases, have enlarged active sites that can accommodate a range of DNA lesions, including thymine dimers, abasic sites, and other DNA modifications (Friedberg et al., 2002, Goodman and Woodgate, 2013). However, our data demonstrate that elimination of the translesion polymerases (encoded by *dinB*, *polB*, or *umuDC*) does not increase DarT^G49D^ toxicity (Figure 4A). The second pathway that can restore DNA replication in the presence of damaged forks is HR. The repair of broken forks and double-stranded breaks (mediated by RecBC) and RecF-dependent recombinational gap filling are both RecA dependent (Persky and Lovett, 2008), consistent with our finding that RecA has a profound effect in preventing DarT^G49D^ toxicity beyond its role in inducing the SOS response (Figures 3C and 4B). Although loss of *recD* did not alter sensitivity to DarT^G49D^ (Figure 4C), this protein is not essential for recombination through RecBC (Lovett et al., 1988). Therefore, we cannot exclude a possible contribution of the broken fork or double-stranded break repair pathways in removing ssDNA ADP-ribosylation (Figure 7). However, deletion of *recF* markedly decreased bacterial viability following *darT*^G49D^ expression (Figure 4D). Although RecF-mediated HR can promote recombinational repair of double-stranded breaks in a *sbcB* mutant (suppressor of *recBC*) (Kolodner et al., 1985), RecF can also contribute to the repair of SSGs (Spies and Kowalczykowski, 2005). SSGs are usually generated when non-coding lesions block DNA replication of the affected strand, whereas replication of the undamaged strand extends beyond the lesion (Spies and Kowalczykowski, 2005). Based on our data, we hypothesize that ADP-ribosylation targets ssDNA loops during DNA replication. For instance, initiation of Okazaki fragment synthesis from a new primer downstream of the lesion leaves an SSG that is filled via RecF-mediated HR.

RecF-mediated HR does not eliminate ADPr from ssDNA but is likely to transfer the lesion to duplex DNA. Therefore, if the adduct is not removed before the next round of DNA replication, the dsDNA ADPr continues to pose a potent threat to genome integrity. Although the discontinuous nature of lagging strand synthesis permits lesion skipping, blocks on the leading strand likely involve more complex replication restart mechanisms (Yeeles and Marians, 2013), requiring removal of ADPr groups from dsDNA. Our single-molecule tracking assays and genetic dissection indicate that NER performs this function, acting after RecF-mediated HR to remove ADPr from dsDNA (Figures 6A and 6C), although we cannot exclude a role for BER because of the redundant nature of this pathway. Importantly, live-cell imaging demonstrated that activation of NER is RecF-dependent following DarT expression, but not after exposure to UV light. These results are consistent with previous reports of links between RecF and NER, during repair following UV-induced damage (Courcelle et al., 1999), and when replication has been stalled by an N‐2‐acetylaminofluorene adduct on guanine (Bichara et al., 2007). NER localizes and excises bulky DNA lesions that cause a structural distortion of the DNA helix (Liu et al., 2011). Lesions dealt with by NER vary in their chemical and structural composition, from abasic sites to polycyclic aromatic hydrocarbon adducts or even to protein-DNA crosslinks of up to 10 kDa (Nakano et al., 2007, Van Houten et al., 2005). An ADPr-modified base is probably a substrate for NER, considering its significant size (consisting of a ribose sugar linked to ADP) could introduce distortions in the DNA helix. Strains lacking *recF* are significantly more sensitive to DarT^G49D^ toxicity than those lacking *uvrA* (Figures 4D and 5C). This suggests that another pathway or pathways may contribute to the removal of dsDNA ADP-ribosylation. Our data clearly rule out a role of MMR in this context (Figure 5A). However, although we found no evidence that BER counteracts DarT toxicity (Figure 5B), we cannot exclude this possibility because of redundancy of this repair pathway. Although we saw no increase in sensitivity to DarT following inactivation of both AP endonucleases, bifunctional glycosylases can incise the DNA backbone during BER (Krokan and Bjørås, 2013).

In conclusion, we show that DNA ADP-ribosylation is perceived as DNA damage, with the SOS response acting to prevent DarT toxicity. Removal of ADPr from DNA requires the striking sequential activity of two DNA repair pathways to convert a single-stranded lesion into a double-stranded lesion via RecF-mediated HR, before being removed from the duplex by NER. Our results provide insights into how this DNA modification is dealt with in cells. The conservation of DNA ARTs, RecF-mediated homologous recombination, and NER in eukaryotes (including humans) indicates the response to and mechanisms of reversal of DNA ADP-ribosylation we have defined in *E. coli* may well operate in higher organisms.

## STAR★Methods

### Key Resources Table

**Table:** 

REAGENT or RESOURCE	SOURCE	IDENTIFIER
**Antibodies**		

Anti-6X His tag® antibody [HIS.H8]	Abcam	Ab 18184; RRID:AB_423119
Rabbit anti-RecA polyclonal antibody	Abcam	Ab 63797; RRID:AB_1142554
Goat anti-rabbit IgG HRP conjugate secondary antibody	Santa Cruz	sc-2357
Goat anti-mouse IgG HRP conjugate secondary antibody	DAKO	Cat#P044701-2
Mouse anti-BrdU monoclonal antibody	BD Biosciences	Cat#347580, B44 clone; RRID:AB_400326
Mouse anti-ssDNA antibody	Developmental Studies Hybridoma Bank, University of Iowa	N/A

**Bacterial and Virus Strains**		

Enteropathogenic *E. coli* isolate O127:H6 str. E2348/69; Str^R^	Knutton et al., 1987	N/A
BL21(DE3) *E. coli* expression strain: F^-^*omp*T *hsd*S(r_B_^-^ m_B_^-^) *dcm*^+^ Tet^r^*gal* λ(DE3) *end*A [*arg*U *pro*L]	Agilent (previously stratagene)	Cat#200131
Dh5α (*fhuA*2 Δ(*argF*-*lacZ*) *U169 phoA glnV44* Φ*80* Δ(*lacZ*)*M15 gyrA96 recA1 relA1 endA1 thi-1 hsdR17*)	Hanahan, 1983	N/A
SM10 (*thi thr leu tonA lacy supE recA*:*:RP4-2-Tc::Mu* λpir; *kan*^R^)	Simon et al., 1983	N/A
*E. coli* λpir (λpir Δ*dapA* Δ*recA; apra*^R^*erm*^R^*zeo*^R^)	Herrero et al., 1990	N/A
BTH101 (*F- cya-99 araD139 galE15 galK16 rpsL1 (Strr) hsdR2 mcrA1 mcrB1*)	Karimova et al., 2000	N/A
See Table S2 for additional strains generated in this study		N/A

**Oligonucleotides**		

See Table S1 for oligonucleotides used for ADP-ribosylation assays		N/A
See Table S3 for primers used for cloning		N/A

**Recombinant DNA**		

pCONJ4S (R6Kγ origin in a pKNG101 with *sacB* and sf*gfp; ampR*^*)*^	Kaniga et al., 1991	N/A
pBAD33 (Medium copy plasmid with an arabinose-inducible promoter; *cm*^R^)	Guzman et al., 1995	N/A
pET28A (Medium copy plasmid containing the IPTG-inducible promoter; *kan*^R^)	Novagen	Cat#69864-3
pUC19 (High copy plasmid; *amp*^R^)	NEB	Cat#N3041S
pMR101 (Low copy plasmid; *kan*^R^)	This paper	N/A
pUT18 (Vector encoding T18 fragment of *B. pertussis cyaA*; *amp*^R^)	Karimova et al., 1998	N/A
pKT25 (Vector encoding T25 fragment of *B. pertussis cyaA*; *cm*^R^)	Karimova et al., 1998	N/A
pBAD33_*darT*^G49D^ (pBAD33 carrying EPEC *darT*^G49D^; *cm*^R^)	This paper	N/A
pBAD33_*darT*^E170A^ (pBAD33 carrying EPEC *darT*^E170A^; *cm*^R^)	This paper	N/A
pET28A_*darG* (pET28A carrying EPEC *darG*; *kan*^R^)	This paper	N/A
pET28A_*darG*^K54A^ (pET28A carrying EPEC *darG*^K54A^; *kan*^R^)	This paper	N/A
pET28A_*macro* (pET28A carrying EPEC macrodomain of *darG* (amino acids 1-113); *kan*^R^)	This paper	N/A
pET28A_*macro*^K54A^ (pET28A carrying EPEC macrodomain of *darG*^K54A^ (amino acids 1-113); *kan*^R^)	This paper	N/A
pET28A_*Cterm* (pET28A carrying EPEC C-terminal domain of *darG* (amino acids 1-16 + 112-330); *kan*^R^)	This paper	N/A
pMR101_*recA* (pMR101 carrying EPEC *recA* under control of its endogenous promoter; *kan*^R^)	This paper	N/A
pMR101_*recF* (pMR101 carrying EPEC *recF* under control of its endogenous promoter; *kan*^R^)	This paper	N/A
pMR101_*recG* (pMR101 carrying EPEC *recG* under control of its endogenous promoter; *kan*^R^)	This paper	N/A
pMR101_*ruvAB* (pMR101 carrying EPEC *ruvAB* under control of its endogenous promoter; *kan*^R^)	This paper	N/A
pMR101_*ruvC* (pMR101 carrying EPEC *ruvC* under control of its endogenous promoter; *kan*^R^)	This paper	N/A
pUT18_*zip* (pKT18 carrying leucine zipper from the yeast transcriptional activator GCN4)	Karimova et al., 1998	N/A
pUT18_*darG* (pKT18 carrying EPEC *darG*; *amp*^R^)	This paper	N/A
pUT18_*macro* (pKT18 carrying EPEC macrodomain of *darG* (amino acids 1-113); *amp*^R^)	This paper	N/A
pUT18_*Cterm* (pKT18 carrying EPEC C-terminal domain of *darG* (amino acids 1-16 + 112-330); *amp*^R^)	This paper	N/A
pKT25_*darT*^G49D^ (pKT25 carrying EPEC *darT*^G49D^; *amp*^R^)	This paper	N/A
pKT25_*zip* (pKT25 carrying leucine zipper derived from the yeast transcriptional activator GCN4)	Karimova et al., 1998	N/A

**Software and Algorithms**		

ImageJ	Schneider et al., 2012	https://imagej.nih.gov/ij/
Prism - GraphPad	GraphPad Software, Inc. Accessed 5 November 2008	https://www.graphpad.com/scientific-software/prism/
MATLAB	MATLAB, 2013; MATLAB, 2016	https://www.mathworks.com/products/matlab.html

### Lead Contact and Materials Availability

Further information and requests for resources and reagents should be directed to and will be fulfilled by the Lead Contact, Christoph M. Tang (christoph.tang@path.ox.ac.uk).

#### Materials Availability Statement

All strains and plasmids generated from this study are available from the Lead Contact without restriction.

### Experimental Model and Subject Details

All *E. coli* or EPEC strains were cultivated in lysogeny broth (LB) or in M9 minimal medium, at 37°C. Experiments were performed with mid-log phase growing bacteria.

### Method Details

#### Bacterial strains and media

*E. coli* strains used in this study are listed in Table S2. *E. coli* was grown at 37°C in lysogeny broth (LB, Invitrogen) or in M9 minimal medium (M9 minimal salts with 47.76 mM Na_2_HPO_4_, 22.04 mM KH_2_PO_4_, 8.56 mM NaCl and 18.69 mM NH_4_Cl), 100 μM CaCl_2_, 2 mM MgSO_4_, 50x MEM amino acids (Sigma-Aldrich), 100X MEM vitamins (Sigma-Aldrich) and 1 mM L-proline). Antibiotics and inducers were added as required at the following final concentrations: carbenicillin, 100 μg/mL; chloramphenicol, 5 μg/mL; kanamycin, 50 μg/mL; arabinose, 0.8% (w/v); glucose, 0.8% (w/v); DAP (2,6-diaminopimelic) which is essential for growth of *E. coli* λpir (Ferrières et al., 2010), 300 μM; IPTG (isopropyl β-D-1-thiogalactopyranoside), 50 μM.

#### Genetic manipulations

Constructs were assembled into plasmids using NEBuilder HiFi DNA Assembly master mix (New England Biolabs) before being transformed into *E. coli* DH5α or *E. coli* SM10λ*pir*. For generating mutants, plasmids were mobilized from *E. coli* λ*pir* into *E. coli* BL21(DE3) or EPEC by conjugation. Mutants were checked by PCR (with primers hybridizing on chromosomal DNA around 700 bp from either side of the deleted gene), followed by sequencing. Plasmids for complementation were electroporated into *E. coli*. Strains for PALM experiments were obtained using P1 phage transduction. Primers used are listed in Table S3.

#### Toxicity assays

To assess the function of DarTG, *darT* variants were expressed under the control of an arabinose-inducible promoter in pBAD33 while *darG* variants were cloned under the control of an IPTG-inducible promoter in pET28A. Bacteria containing both plasmids were grown in LB supplemented with glucose at 37°C overnight, and diluted to an optical density (OD_600nm_) of 0.01 then allow to grow to an OD_600nm_ of 0.1; cells were then pelleted and re-suspended in LB containing arabinose and IPTG to induce *darT* and *darG* expression, respectively. At time points post-induction, the number of viable bacteria were determined by plating a serial dilution of cultures onto solid medium containing glucose and IPTG. Plates were incubated at 37°C overnight.

#### UV sensitivity

To assess the bacterial survival following UV exposure, bacteria were grown in LB at 37°C overnight, and diluted to an optical density (OD_600nm_) of 0.01 then allow to grow to an OD_600nm_ of 0.4. Serial dilutions of cultures were spotted onto LB agar plates before exposing bacteria to the appropriate UV dose, using a Stratalinker UV crosslinker (1200 J). Plates were incubated at 37°C overnight.

#### Western blot analysis

Bacteria were grown as for viability assays. After induction of *darT* variants for 3 hours, cell lysates were prepared and the amount of proteins were normalized based on the OD_600nm_ of cultures. Bacteria were pelleted, re-suspended in SDS-polyacrylamide gel electrophoresis (PAGE) loading buffer (100 mM Tris-HCl pH 6.8, 20 μM *β-*mercaptoethanol, 4% (w/v) SDS, 0.2% (w/v) bromophenol blue, 20% (v/v) glycerol) and boiled for 10 minutes. Samples were resolved on 12% SDS-PAGE gels and electrotransferred to nitrocellulose membranes. Membranes were blocked in 5% (w/v) milk-PBS with 0.05% (v/v) Tween 20 (PBS-T) for 1 hour, then incubated with a mouse anti-His monoclonal antibody (1:1,000; Abcam Cambridge 18184, overnight incubation at 4°C), or a rabbit anti-RecA polyclonal antibody (1:10,000; Abcam Cambridge 63797; 1 hour at room temperature). Goat anti-mouse (DAKO, P0447) or goat anti-rabbit (Santa Cruz, sc-2004) IgG HRP conjugate secondary antibody was used at a final concentration of 1:10,000 in 5% milk-PBS-T for 1 hour at room temperature. Membranes were washed 3 times for 5 minutes in PBS-T and binding was detected using ECL western blotting detection kit (Amersham). Bands intensities were measured by selecting relevant areas on blots and measuring pixels with ImageJ software (https://imagej.nih.gov/ij/).

#### BrdU incorporation assay

Bacteria were grown as above and at different time points post-induction, samples were taken, normalized to an OD_600_ 1.5, pelleted and then re-suspended in 5 mL of LB with 0.8% (w/v) arabinose, 20 μM BrdU (5-bromo-2′-deoxyuridine; Sigma-Aldrich) and 33 nM thymidine (Sigma-Aldrich). Cells were incubated at 37°C for 45 minutes before being pelleted and the supernatant removed. The pellet was frozen in dry ice. Genomic DNA was extracted using the Wizard Genomic DNA purification kit (Promega), and quantified with Qubit dsDNA HS Assay kit (Life Technologies). The DNA concentration was adjusted to 10 ng/μl, and 10 μL of DNA was denatured by the addition of 1 μL 4 M NaOH for 20 minutes at room temperature, then neutralized with 11 μL of 1M Tris-HCl pH 6.8 on ice. Denatured ssDNA (3 μL or 5 μl) was spotted onto nitrocellulose membranes (Amersham - Potran 0.45 μm) and fixed using a Stratalinker UV crosslinker (1200 J). Membranes were blocked with 5% milk-PBS-T for 1 hour at room temperature and washed 3 times with PBST.

Incorporation of BrdU was detected by using an anti-BrdU antibody (B44 clone, BD Biosciences, final concentration of 1:1,000) in 0.5% milk-PBS-T incubated overnight at 4°C. As a loading control, an anti-ssDNA antibody was used (from the Developmental Studies Hybridoma Bank, University of Iowa, final concentration of 1:200) in 5% milk-PBS-T incubated overnight at 4°C. Membranes were washed 3 times with PBS-T. Goat anti-mouse IgG HRP conjugate secondary antibody (DAKO, P0447) was used at a final concentration of 1:10,000 in 0.5% milk-PBS-T for 1 hour at room temperature. The membranes were washed 3 times with PBS-T and detection was performed using ECL western blotting detection kit (Amersham). DNA replication was determined as the intensity of BrdU signal/intensity of ssDNA.

#### ADP-ribosylation and de-ribosylation assays

Purified DarT and DarG versions were obtained by *in vitro* transcription translation (IVTT) using ExpressWay Cell-Free *E. coli* Expression System (Life Technologies). *In vitro* transcribed translated proteins (5 μl) were incubated with 10 μM of a single-stranded oligonucleotide and 5 μM ^32^P-NAD^+^ (80 kBq/reaction specific activity) in a final reaction volume of 20 μL containing ADP-ribosylation buffer (50 mM Tris-HCl pH 8, 150 mM NaCl) and 10 mM EDTA for 60 minutes at 30°C. Reactions were stopped by the addition of 2X ULD buffer (8 M urea, 20 mM EDTA, 2 mM Tris-HCl pH 7.5) then incubated at 94°c for 3 minutes. When appropriate, samples were diluted with ADP-ribosylation and 2X ULD buffer prior to analysis by denaturing PAGE.

A single-stranded oligonucleotide with a single modification site (GJ_S58: GGCCCGCCGTTTC) or double-stranded DNA (GJ_S58 annealed with 1.25x excess of GJ_S58rc: GAAACGGCGGGCC) at 10 μM were subjected to ADP-ribosylation reaction (as above) and analyzed by thin-layer chromatography. Briefly, 1 μl of the reaction was spotted on PEI cellulose plates (Macherey-Nagel), allowed to air dry and were developed in 0.25 M LiCl and 0.25 M formic acid. The plate was dried and exposed to autoradiography films (Kodak Carestream BioMax MS).

For non-radioactive assays, *in vitro* transcribed translated proteins (1:5) were incubated with 4X ADP-ribosylation buffer (50 mM Tris-HCl pH 8, 150 mM NaCl), 50 μM NAD^+^, 10 mM EDTA and 10 μM of single-stranded oligonucleotide (Table S1) for 30 minutes at 30°C. Reactions were stopped by addition of 2X ULD buffer then incubated at 94°c for 3 minutes.

To assess de-ADP-ribosylation, ADP-ribosylated oligonucleotides were obtained by incubating ADPr-EL1 oligonucleotide (Table S1) with DarT, PAGE purified and desalted in 10 mM Tris-HCl pH 7.5. Purified DarG variants obtained by *in vitro* transcription/translation (1:25) were incubated with 1 μM of ADP-ribosylated oligonucleotide, 10 mM Tris-HCl pH 7 and 5 mM EDTA. Reactions were incubated at 37°C for 30 minutes before being stopped with a 2X ULD buffer and incubated at 94°C for 3 minutes.

All the reactions were run on 15% polyacrylamide (25:1) 8 M urea gels in 1X TBE buffer (89 mM Tris pH 8, 89 mM boric acid, 2 mM EDTA). For non-radioactive assays, gels were washed in 1X TBE buffer for 5 minutes, then incubated for 5 minutes in ethidium bromide solution. Gels were washed with ddH_2_O then visualized with a UVP BioDoc-It system. For radioactive assays, gels were dried and exposed to radiography films Carestream Kodak BioMax MS film for 3 hours.

#### Bacterial two hybrid assay

The interaction between DarT and DarG was detected as described previously (Karimova et al., 1998). *E. coli* BTH101 was transformed with vectors expressing T18 and T25 fusions, and grown at 37°C overnight in LB. Bacteria were diluted to OD_600_ 0.1 and incubated at 37°C until the OD_600_ 0.4 was reached. Cell lysates were generated with Pop culture (Novagen) and 1 unit of r-lysozyme (Novagen). A 15 μL of aliquot lysates was mixed with 135 μL of Z buffer (60 mM Na_2_HPO_4_, 40 mM NaH_2_PO_4_, 10 mM KCl, 1 mM MgSO_4_, 50 mM β-mercaptoethanol adjusted to pH 7) and 30 μL of fresh 13.28 mM ONPG (ortho-nitrophenyl-β-galactoside) diluted in 100 mM phosphate buffer (60 mM Na_2_HPO_4_, 40 mM NaH_2_PO_4_, pH 7). The reaction was incubated at room temperature until the color turned yellow and was stopped by adding 50 μL of 1M Na_2_CO_3_. The absorbance was then measured at OD_420nm_ and the Miller units were determined as previously (Miller, 1992).

#### Single-molecule tracking by PALM

Single-molecule tracking PALM in live *E. coli* BL21(DE3) was performed as previously described (Banaz et al., 2019, Stracy et al., 2016). Bacteria were grown in M9 minimal medium supplemented with 0.2% (w/v) glucose and incubated at 37°C overnight. Overnight bacterial cultures were diluted to OD_600_ 0.01 in M9 medium with 0.2% (w/v) glucose and incubated at 37°C until the OD_600_ reached 0.3. Bacteria were washed 3 times in 1 mL of M9 medium containing 0.2% (w/v) arabinose, incubated at 37°C for 15 minutes before spinning down and re-suspending the cells in a few μl. Bacteria were spotted on M9 0.2% arabinose 0.1% agar pads and imaged using a custom-built total internal reflection fluorescence microscope. Photoactivable mCherry was activated using a 405 nm laser and excited at 561 nm. Single UvrB-PAmCherry tracking was performed by taking a 10,000 frames movie, with an interval of 15 millisec between each frame. Immobile and mobile UvrB-PAmCherry were distinguished based on the mean-squared displacement for each track with 4 steps at Δ*t* (interval of time) = 15 millisec, and calculating the apparent coefficient diffusion D^∗^ = MSD/(4 Δ*t*) as done previously (Stracy et al., 2016). The number of tracks corresponds to the tracks with a minimum of 4 steps. The average duration of tracks was 2.5 frames. Approximately 10% of tracks lasted for 5 or more frames (i.e., > 4 steps). Shorter tracks were not included in the diffusion analysis. The diffusion coefficient of immobile UvrB-PAmCherry was identified as shown in Figure S5C.

### Quantification and Statistical Analysis

The number of biological replicates and/or the number of cells are/is indicated in the legend for each experiment, with SD included on each plot. Values for toxicity assays were first log-transformed before being analyzed. Differences were assessed using Student’s t test, unpaired t test (two-tailed) or two-way ANOVA as appropriate (see figure legends) in GraphPad Prism software (GraphPad). P values correlate with symbols as follows: ns = not significant, p > 0.05, ^∗^ p ≤ 0.05, ^∗∗^ p ≤ 0.01, ^∗∗∗^ p ≤ 0.001, ^∗∗∗∗^ p ≤ 0.0001.

### Data and Code Availability

There is no dataset/code associated with the paper.

## Supplemental Information

Document S1. Figures S1–S7 and Tables S1 and S2

Table S3. Primers Database, Related to STAR Methods

Document S2. Article plus Supplemental Information
