# Supplementary material for: DNA ADP-Ribosylation Stalls Replication and Is Reversed by RecF-Mediated Homologous Recombination and Nucleotide Excision Repair
Source: Cell Rep. 2019 Feb 4;30(5):1373–1384.e4. doi: 10.1016/j.celrep.2020.01.014 (PMC7003065; doi:10.1016/j.celrep.2020.01.014)
Supplement: Document S1. Figures S1–S7 and Tables S1 and S2 [file mmc1.pdf]

Cell Reports, Volume *30*

Supplemental Information

DNA ADP-Ribosylation Stalls Replication and Is Reversed by RecF-Mediated Homologous Recombination and Nucleotide Excision Repair

Emeline Lawarée, Gytis Jankevicius, Charles Cooper, Ivan Ahel, Stephan Uphoff, and Christoph M. Tang

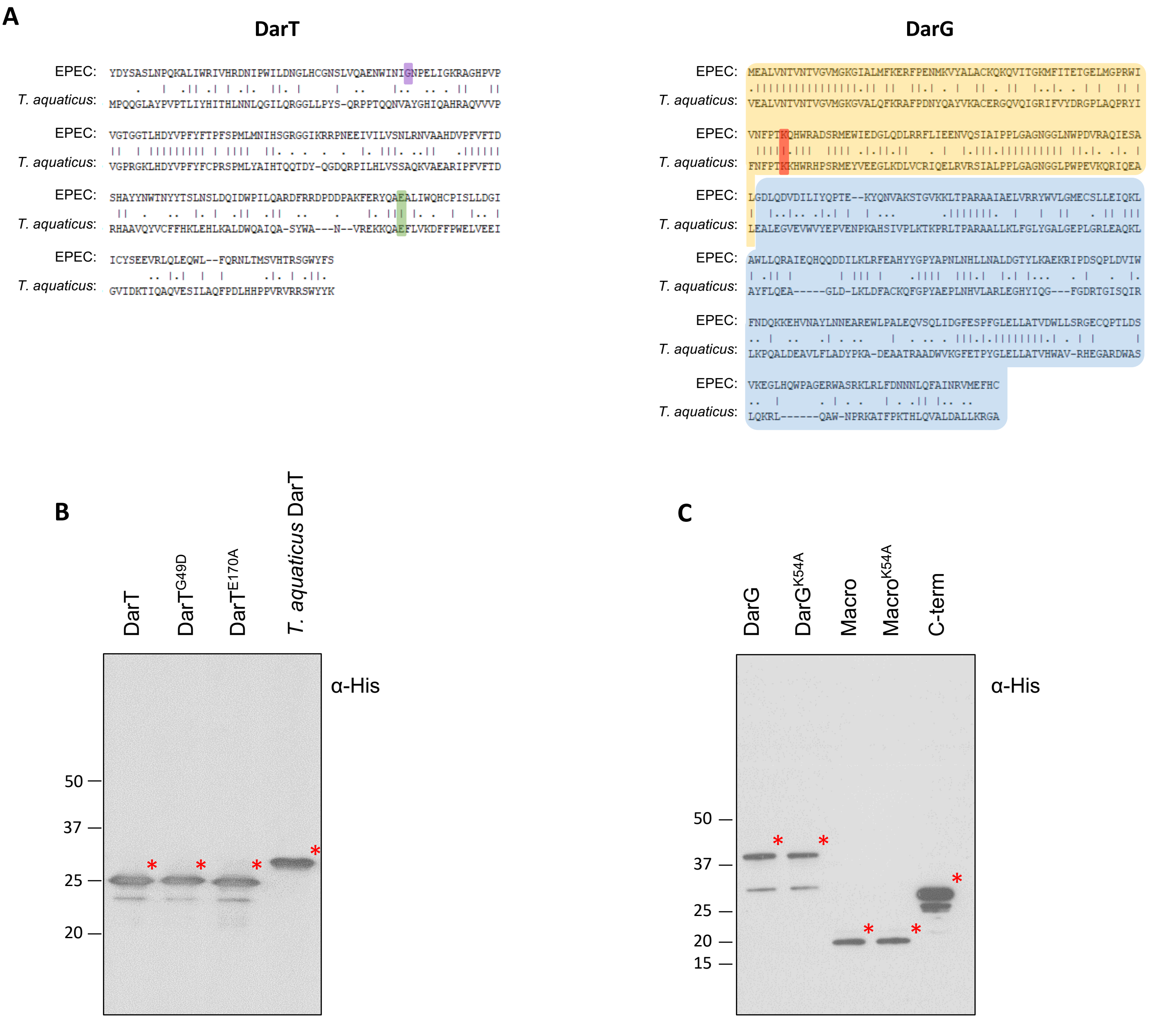

Figure S1: Alignment of DarTG from *T. aquaticus* and EPEC, and the corresponding purified proteins after *in vitro* transcription/translation, related to Figures 1 and 2. **(A)** The amino acid similarity between *T. aquaticus* and EPEC DarT or DarG is 47% and 59%, respectively. Identical residues are shown with a line, similar residues are shown with a dot. The mutated glycine in EPEC DarT (G49D) is shown in purple while the mutated glutamic acid residues (DarT^{E160A} in *T. aquaticus* and DarT^{E170A} in EPEC) are marked in green. The macrodomain of DarG is highlighted in yellow while the C-terminal domain of DarG is showed in blue. The catalytic lysine residues of DarG (K80A in *T. aquaticus* and K54A in EPEC) are marked in red. Alignment was performed by Clustal O with JalView software. Levels of His-tagged **(B)** DarT and **(C)** DarG variants (indicated with red asterisks) obtained after *in vitro* transcription/translation determined by Western blot analysis using an anti-His antibody.

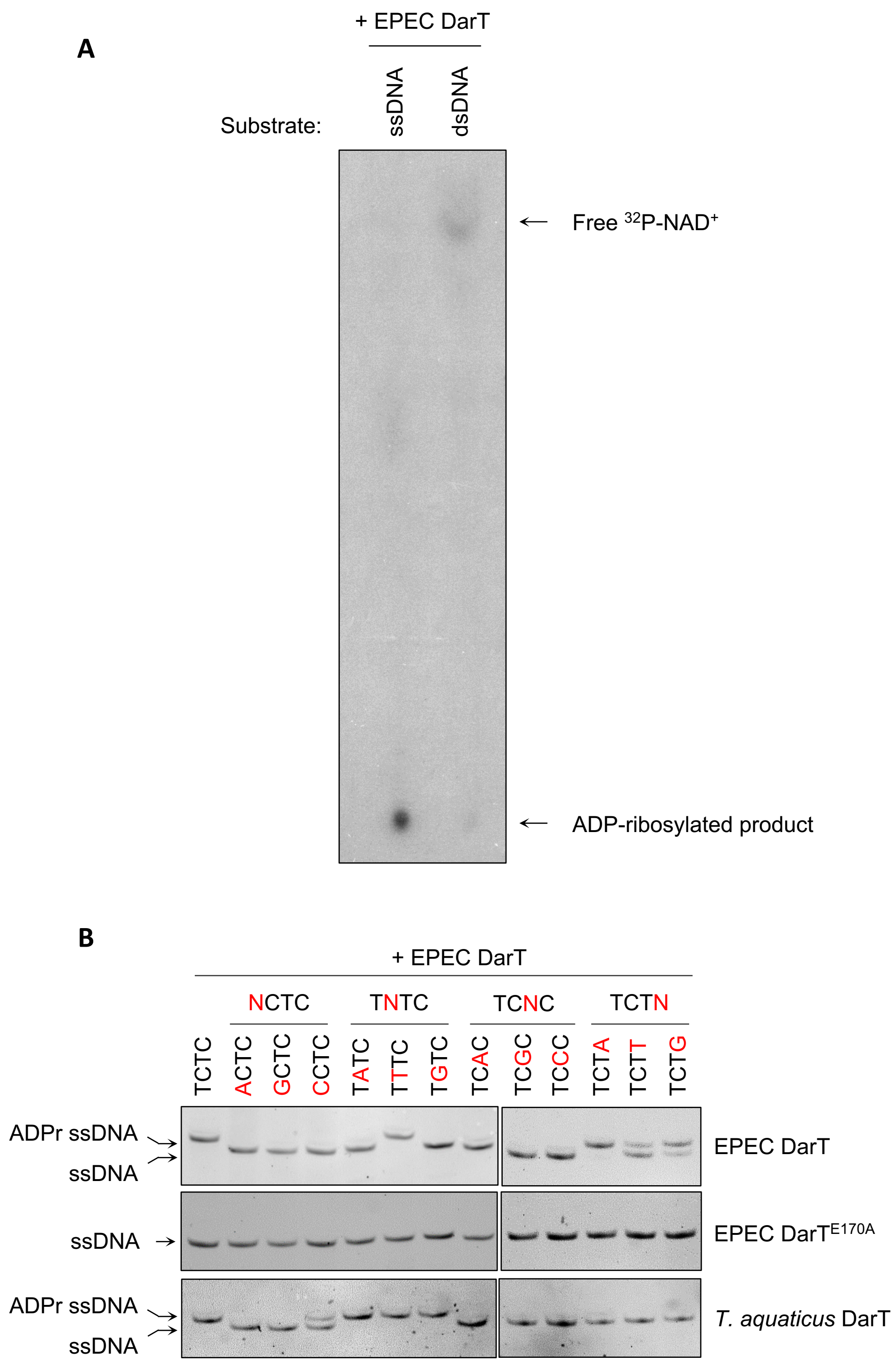

Figure S2: EPEC DarT only ADP-ribosylates ssDNA, on a different DNA sequence than *T. aquaticus* DarT, related to Figure 1. **(A)** ADP-ribosylation of 13-mer ssDNA or dsDNA oligonucleotides incubated with EPEC DarT with ^{32P}NAD^{+} and separated by thin-layer chromatography. Sequences of the oligonucleotides are provided in the Star methods. **(B)** ADP-ribosylation of 27-mer ssDNA oligonucleotides incubated with purified EPEC DarT or DarT^{E170A} or *T. aquaticus* DarT. The region with different sequence is shown above each lane. ADPr ssDNA: ADP-ribosylated ssDNA. All oligonucleotides are listed in Table S1. n = 3, representative data from one experiment shown.

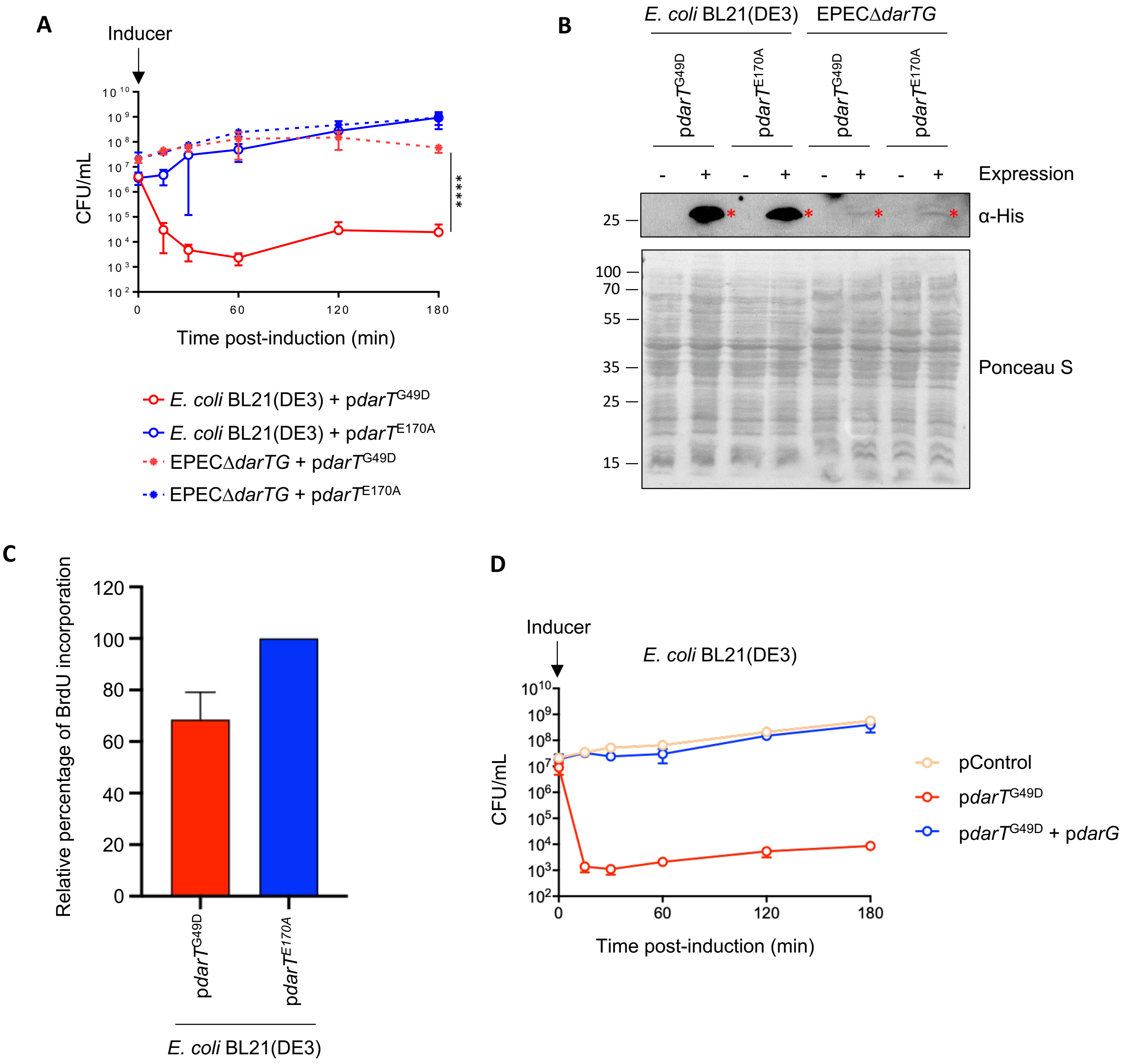

Figure S3: Differential DarT^{G49D}/DarT^{E170A} toxicity and expression levels in *E. coli* BL21(DE3) and EPEC, where DarT^{G49D} inhibits DNA replication in *E. coli* BL21(DE3) within 5 min of expression, related to Figures 1 and 5. **(A)** Viability of *E. coli* BL21(DE3) and EPECΔ*darTG* following expression of *darT*^{G49D} or *darT*^{E170A}. n = 3 ± s. d., **** $p < 0.0001$ by two-way ANOVA between *E.coli* BL21(DE3) + p*darT*^{G49D} and EPECΔ*darTG* + p*darT*^{G49D}. **(B)** Levels of His-tagged DarT^{G49D} or DarT^{E170A} (red asterisks) in cell lysates of *E. coli* BL21(DE3) and EPECΔ*darTG* using an anti-His antibody. Expression of His-DarT^{G49D}/His-DarT^{E170A} is repressed by addition of 0.8% glucose or expressed by the addition 0.8% arabinose for 3 hrs. Ponceau S staining is shown as the loading control. n = 3, representative data from one experiment shown. **(C)** Relative values were obtained by calculating the ratio of the amount of BrdU incorporated following expression of *darT*^{G49D} vs. *darT*^{E170A}. n = 2 ± s. d. **(D)** Viability of *E. coli* BL21(DE3) grown in M9 medium following expression of *darT*^{G49D} with or without DarG. pControl, empty plasmid; n = 2 ± s. d.

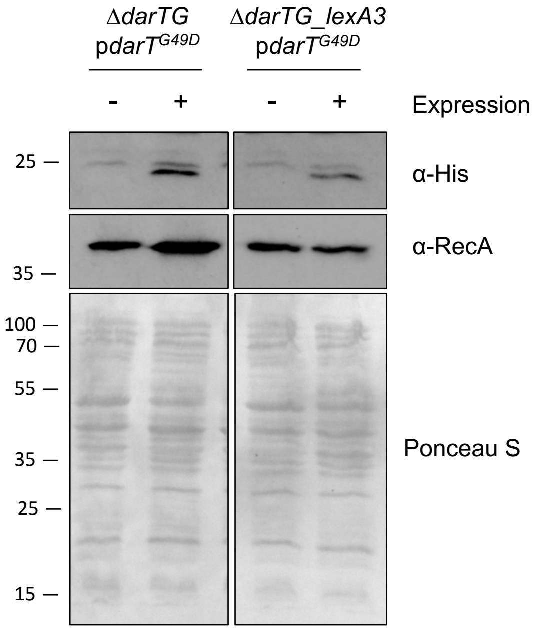

Figure S4: Cellular RecA levels do not increase in the absence of an SOS response, related to Figure 3. Western blot analysis of RecA expression levels in cell lysates of EPECΔ*darTG* and EPECΔ*darTG_lexA3* using an anti-His (to detect levels of His tagged $DarT^{G49D}$ or $DarT^{E170A}$) and anti-RecA antibody after 3 hours of *darT*^{G49D} expression. Ponceau S staining is shown as the loading control. n = 3, representative data from one experiment shown.

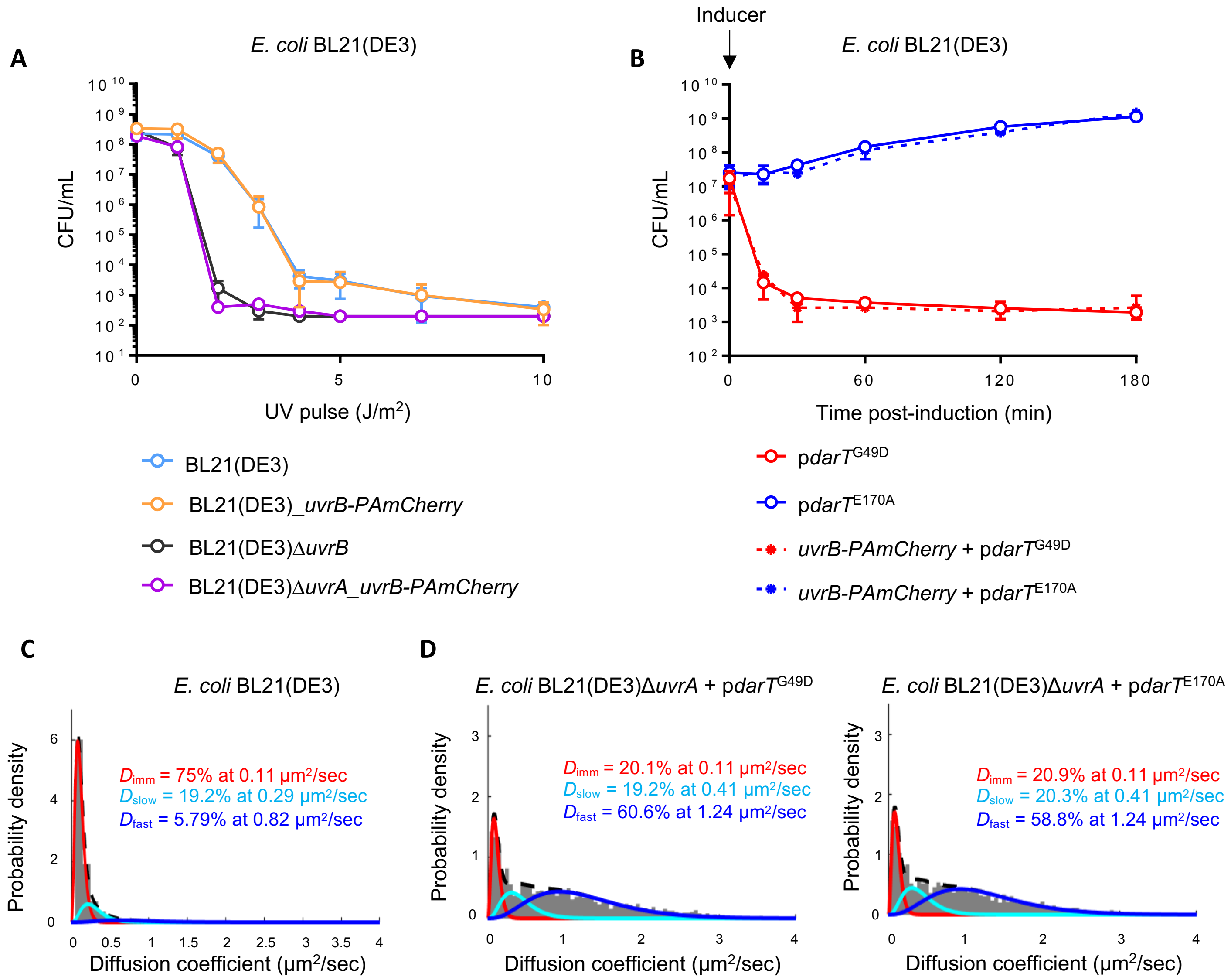

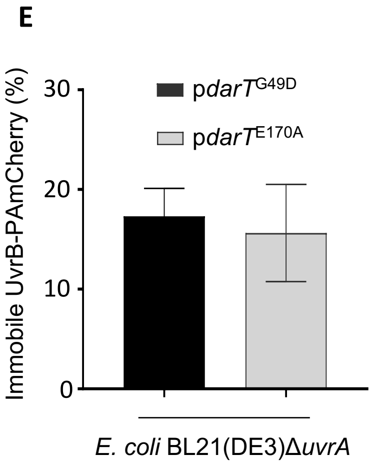

Figure S5: UvrB fused to PAmCherry is functional, does not affect bacterial viability following UV exposure and UvrB-PAmCherry recruitment does not occur in a Δ*uvrA* strain, related to Figure 5. **(A)** Viability of *E. coli* BL21(DE3), *E. coli* BL21(DE3)_*uvrB-PAmCherry*, *E. coli* BL21(DE3)Δ*uvrB* and *E. coli* BL21(DE3)Δ*uvrA_uvrB-PAmCherry* after UV exposure. n = 3 ± s. d. **(B)** Viability of *E. coli* BL21(DE3) and *E. coli* BL21(DE3)_*uvrB-PAmCherry* following expression of $darT^{G49D}$ or $darT^{E170A}$. n = 3 ± s. d. **(C)** To determine the immobile D*, cells were fixed with 3% paraformaldehyde to crosslink UvrB-PAmCherry molecules to DNA (and so render them immobile). Using unconstrained fitting, the calculated immobile D* of UvrBPAmCherry is 0.11 μm2/sec. This value was used as a constraint in the fit to measure the percentage of immobile UvrBPAmCherrymolecules in live cells with all three species (immobile, slow, fast) present. **(D)** Determination of D* values of UvrB-PAmCherry in *E. coli* BL21(DE3)Δ*uvrA*, fitted with a three constrained species model following 15 min expression of $darT^{G49D}$ or $darT^{E170A}$. **(E)** Percentage of immobile UvrB-PAmCherry in *E. coli* BL21(DE3)Δ*uvrA* after 15 min expression of $darT^{G49D}$ or $darT^{E170A}$. n = 2 biological replicates ± s. d.; total number of cells = 3,698; total number of tracks = 38,918.

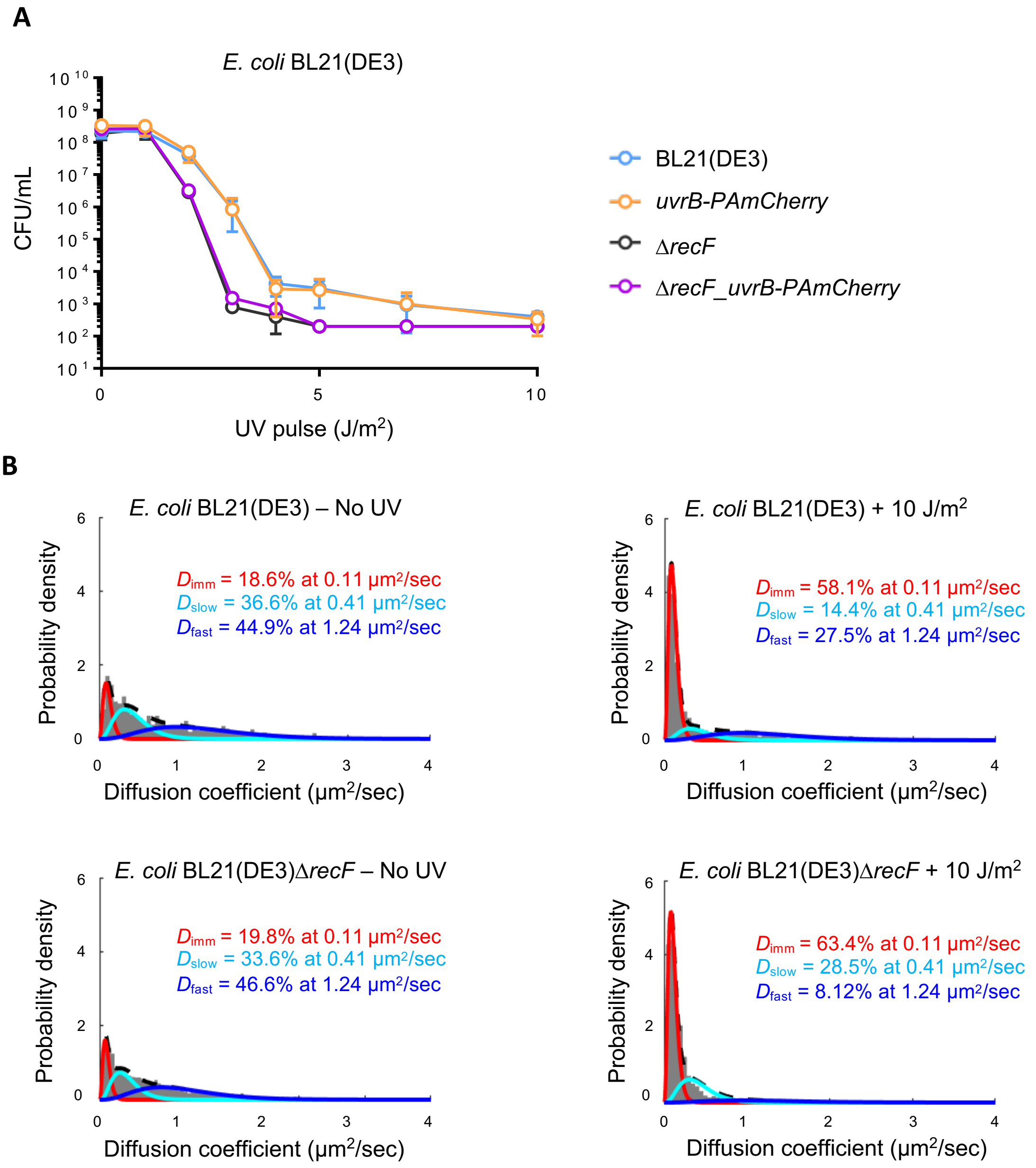

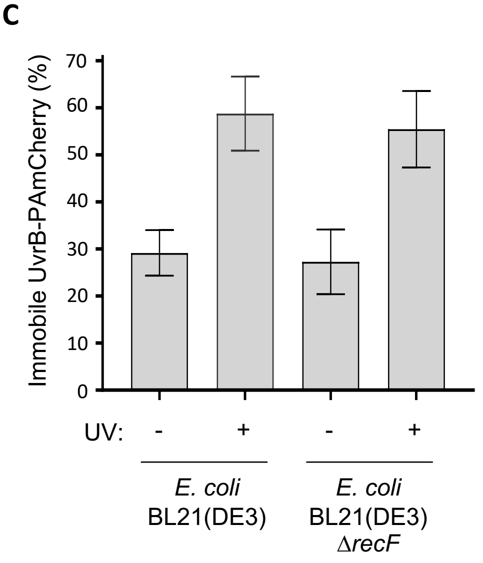

Figure S6: NER is functional in a Δ*recF* strain, related to Figure 6. **(A)** Viability of BL21(DE3), BL21(DE3)_*uvrB-PAmCherry*, BL21(DE3)Δ*recF* and BL21(DE3)Δ*recF_uvrB-PAmCherry* after exposure to different UV doses. n = 3 ± s. d. **(B)** Determination of D* values of UvrB-PAmCherry in *E. coli* BL21(DE3) and BL21(DE3)Δ*recF*, fitted with a three species model (three constraints fit) with or without UV exposure. **(C)** Percentage of immobile UvrB-PAmCherry in *E. coli* BL21(DE3) and *E. coli* BL21(DE3)Δ*recF* after UV exposure. n = 3 biological replicates ± s. d.; total number of cells = 12,976; total number of tracks = 126,225.

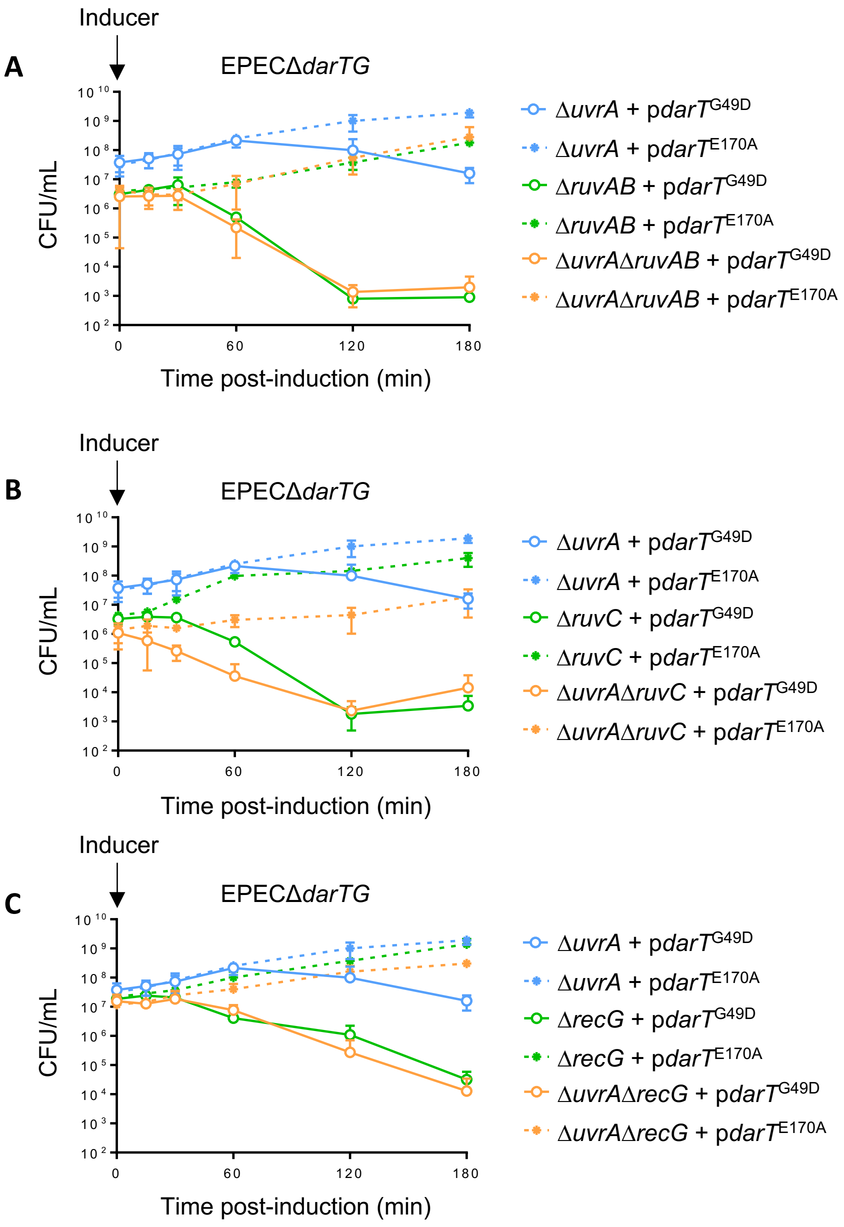

Figure S7: Investigation of the sequentiality between RecF-mediated HR and NER pathways, related to Figure 6. Viability of strains following expression of *darT*^{G49D} or *darT*^{E170A} in EPECΔ*darTG* lacking RecF-mediated HR and/or NER **(A)** EPECΔ*darTG*Δ*ruvAB* and EPECΔ*darTG*Δ*uvrA*Δ*ruvAB*, **(B)** EPECΔ*darTG*Δ*ruvC* and EPECΔ*darTG*Δ*uvrA*Δ*ruvC*, **(C)** EPECΔ*darTG*Δ*recG* and EPECΔ*darTG*Δ*uvrA*Δ*recG*. n = 3 ± s. d. NS = p > 0.9999 by two-way ANOVA between EPECΔ*darTG*Δ*ruvAB*/Δ*ruvC*/Δ*recG* + p*darT*^{G49D} and EPECΔ*darTG*Δ*uvrA*Δ*ruvAB*/Δ*ruvC*/Δ*recG* + p*darT*^{G49D}.

Name	Sequence
ADPr-EL1	GAGCTGTACAAGTCAGATCTCGAGCTC
ADPr-EL2	GAGCTGTACAAGTCAGAACTCGAGCTC
ADPr-EL3	GAGCTGTACAAGTCAGAGCTCGAGCTC
ADPr-EL4	GAGCTGTACAAGTCAGACCTCGAGCTC
ADPr-EL5	GAGCTGTACAAGTCAGATATCGAGCTC
ADPr-EL6	GAGCTGTACAAGTCAGATTTCGAGCTC
ADPr-EL7	GAGCTGTACAAGTCAGATGTCGAGCTC
ADPr-EL8	GAGCTGTACAAGTCAGATCACGAGCTC
ADPr-EL9	GAGCTGTACAAGTCAGATCGCGAGCTC
ADPr-EL10	GAGCTGTACAAGTCAGATCCCGAGCTC
ADPr-EL11	GAGCTGTACAAGTCAGATCTAGAGCTC
ADPr-EL12	GAGCTGTACAAGTCAGATCTTGAGCTC
ADPr-EL13	GAGCTGTACAAGTCAGATCTGGAGCTC
ADPr-EL14	GAGCTGTACAAGTCAGTTCTCGAGCTC
ADPr-EL15	GAGCTGTACAAGTCAGGTCTCGAGCTC
ADPr-EL16	GAGCTGTACAAGTCAGCTCTCGAGCTC
ADPr-EL17	GAGCTGTACAAGTCAGATCTCTAGCTC
ADPr-EL18	GAGCTGTACAAGTCAGATCTCGAGCTC
ADPr-EL19	GAGCTGTACAAGTCAGATCTCCAGCTC
ADPr-EL20	CTCGACATGATCAGTCGTCTCCTCGAG
ADPr-EL21	CTCGACATGATCAGTCGTTTCCTCGAG

Table S1, related to STAR method**:** Oligonucleotides used for ADP-ribosylation assays

Name	Strain	Relevant genotype or description	Reference
EL131	Dh5α	*fhuA*2 Δ(*argF-lacZ*) *U169 phoA glnV44 Φ80* Δ(*lacZ*)*M15 gyrA96 recA1 relA1 endA1 thi-1 hsdR17*	(Hanahan, 1983)
EL183	SM10	*thi thr leu tonA lacy supE recA*::*RP4-2-Tc::Mu* λpir; *kan*^{R}	(Simon, 1983)
EL615	*E. coli* λpir	λpir Δ*dapA* Δ*recA; apra*^{R} *erm*^{R} *zeo*^{R}	(Herrero et al., 1990)
EL270	BTH101	*F- cya-99 araD139 galE15 galK16 rpsL1 (Strr) hsdR2 mcrA1 mcrB1*	(Karimova et al., 2000)
EL67	BL21(DE3)	*E. coli* expression strain: F$^-$ *omp*T *hsd*S(r_B^- m_B^-) *dcm*$^+$ Tet^r *gal* λ(DE3) *end*A [*arg*U *pro*L]	Stratagene
EL572	BL21(DE3)Δ*recF*	BL21(DE3)Δ*recF*	This study
EL646	BL21(DE3)Δ*uvrA_uvrB-PAmCherry*	BL21(DE3)Δ*uvrA* with chromosomal *uvrB* fused to PAmCherry; Kan^R	This study
EL653	BL21(DE3)Δ*uvrB*	BL21(DE3)Δ*uvrB*	This study
EL325	BL21(DE3)*_uvrB-PAmCherry*	BL21(DE3) with chromosomal *uvrB* fused to PAmCherry; Kan^R	This study
EL614	BL21(DE3)Δ*recF_uvrB-PAmCherry*	BL21(DE3)Δ*recF* with chromosomal *uvrB* fused to PAmCherry; *kan*^{R}	This study
EL1	EPEC	Enteropathogenic *E. coli* isolate O127:H6 str. E2348/69; Str^R	(Knutton et al., 1987)
EL2	EPECΔ*darTG*	EPEC E2348/69Δ*darTG*; Str^R	This study
EL268	EPECΔ*darTG*Δ*dinB*	EPEC E2348/69Δ*darTG*Δ*dinB*; Str^R	This study
EL326	EPECΔ*darTG*Δ*dinB*Δ*polB*Δ*umuD*	EPEC E2348/69Δ*darTG*Δ*dinB*Δ*polB*Δ*umuD*; Str^R	This study
EL377	EPECΔ*darTG*Δ*fpg*	EPEC E2348/69Δ*darTG*Δ*fpg*; Str^R	This study
EL415	EPECΔ*darTG_lexA3*	EPEC E2348/69 containing a point mutation (G85D) in chromosomal *lexA*; Str^R	This study
EL226	EPECΔ*darTG*Δ*mutH*	EPEC E2348/69Δ*darTG*Δ*mutH*; Str^R	This study
EL228	EPECΔ*darTG*Δ*mutS*	EPEC E2348/69Δ*darTG*Δ*mutS*; Str^R	This study
EL378	EPECΔ*darTG*Δ*nei*	EPEC E2348/69Δ*darTG*Δ*nei*; Str^R	This study
EL379	EPECΔ*darTG*Δ*nfo*	EPEC E2348/69Δ*darTG*Δ*nfo*; Str^R	This study
EL269	EPECΔ*darTG*Δ*polB*	EPEC E2348/69Δ*darTG*Δ*polB*; Str^R	This study
EL203	EPECΔ*darTG*Δ*recA*	EPEC E2348/69Δ*darTG*Δ*recA*; Str^R	This study
EL588	EPECΔ*darTG*Δ*recD*	EPEC E2348/69Δ*darTG*Δ*recD*; Str^R	This study
EL526	EPECΔ*darTG*Δ*recF*	EPEC E2348/69Δ*darTG*Δ*recF*; Str^R	This study
EL527	EPECΔ*darTG*Δ*recG*	EPEC E2348/69Δ*darTG*Δ*recG*; Str^R	This study
EL468	EPECΔ*darTG*Δ*ruvAB*	EPEC E2348/69Δ*darTG*Δ*ruvAB*; Str^R	This study
EL528	EPECΔ*darTG*Δ*ruvC*	EPEC E2348/69Δ*darTG*Δ*ruvC*; Str^R	This study
EL307	EPECΔ*darTG*Δ*umuD*	EPEC E2348/69Δ*darTG*Δ*umuD*; Str^R	This study
EL224	EPECΔ*darTG*Δ*uvrA*	EPEC E2348/69Δ*darTG*Δ*uvrA*; Str^R	This study
EL530	EPECΔ*darTG*Δ*uvrA*Δ*recF*	EPEC E2348/69Δ*darTG*Δ*uvrA*Δ*recF*; Str^R	This study
EL531	EPECΔ*darTG*Δ*uvrA*Δ*recG*	EPEC E2348/69Δ*darTG*Δ*uvrA*Δ*recG*; Str^R	This study
EL533	EPECΔ*darTG*Δ*uvrA*Δ*ruvAB*	EPEC E2348/69Δ*darTG*Δ*uvrA*Δ*ruvAB*; Str^R	This study
EL532	EPECΔ*darTG*Δ*uvrA*Δ*ruvC*	EPEC E2348/69Δ*darTG*Δ*uvrA*Δ*ruvC*; Str^R	This study
EL412	EPECΔ*darTG*Δ*xth*	EPEC E2348/69Δ*darTG*Δ*xth*; Str^R	This study

Table S2, related to STAR method **:** Strains used in this study
